# Extended Application of Genomic Selection to Screen Multi-Omics Data for the Development of Novel Pyroptosis-Immune Signatures and Predicting Immunotherapy of Glioma

**DOI:** 10.3389/fphar.2022.893160

**Published:** 2022-05-10

**Authors:** Shuai Ma, Fang Wang, Nan Wang, Jiaqi Jin, Xiuwei Yan, Lili Wang, Xiangrong Zheng, Shaoshan Hu, Jianyang Du

**Affiliations:** ^1^ Department of Neurosurgery, The Second Affiliated Hospital of Zhengzhou University, Zhengzhou, China; ^2^ Emergency Medicine Center, Department of Neurosurgery, Zhejiang Provincial People’s Hospital Affiliated to Hangzhou Medical College, Hangzhou, China; ^3^ Department of Neurosurgery, The Second Affiliated Hospital of Harbin Medical University, Harbin, China; ^4^ Department of Obstetrics and Gynecology, Qilu Hospital of Shandong University, Jinan, China; ^5^ Department of Neurosurgery, Shandong Provincial Hospital Affiliated to Shandong First Medical University, Jinan, China

**Keywords:** pyroptosis-immune signature, tumor immune microenvironment, PIPM risk score, stemness, immunotherapy, pan-cancer

## Abstract

Glioma is one of the most human malignant diseases and the leading cause of cancer-related deaths worldwide. Nevertheless, the present stratification systems do not accurately predict the prognosis and treatment benefit of glioma patients. Currently, no comprehensive analyses of multi-omics data have been performed to better understand the complex link between pyroptosis and immune. In this study, we constructed four pyroptosis immune subgroups by pyroptosis regulators and obtained nine pyroptosis immune signatures by analyzing the differentially expressed genes between the four pyroptosis immune subgroups. Nine novel pyroptosis immune signatures were provided for assessing the complex heterogeneity of glioma by the analyses of multi-omics data. The pyroptosis immune prognostic model (PIPM) was constructed by pyroptosis immune signatures, and the PIPM risk score was established for glioma cohorts with a total of 1716 samples. Then, analyses of the tumor microenvironment revealed an unanticipated correlation of the PIPM risk score with stemness, immune checkpoint expression, infiltrating the immune system, and therapy response in glioma. The low PIPM risk score patients had a better response to immunotherapy and showed sensitivity to radio-chemotherapy. The results of the pan-cancer analyses revealed the significant correlation between the PIPM risk score and clinical outcome, immune infiltration, and stemness. Taken together, we conclude that pyroptosis immune signatures may be a helpful tool for overall survival prediction and treatment guidance for glioma and other tumors patients.

## Graphical Abstract

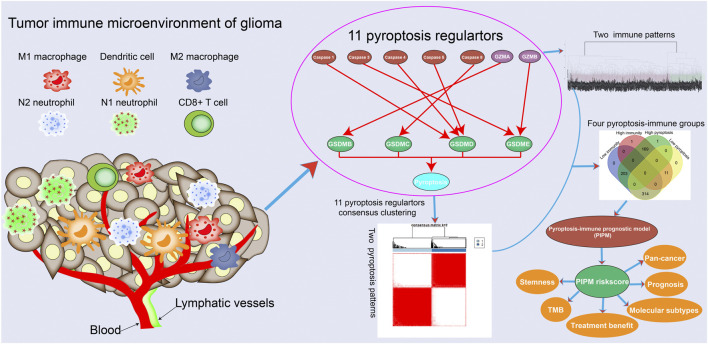

## Introduction

Glioma is the most common and malignant cancer of the central nervous system. Disturbances in the tumor microenvironment (TME) of this disease, which is mainly diagnosed in its advanced stages, may lead to extensive tumor heterogeneity. Moreover, the significant heterogeneity in response to treatment in glioma patients affects the improvement of patient prognosis.

Pyroptosis, a pathway of cleavage and programmed inflammatory cell death distinct from apoptosis, has consequently generated considerable interest among cancer researchers because of the possibility of its clinical applications and overcoming these problems. Furthermore, pyroptosis involves the cleavage of gas proteins through classical and non-classical pathways and can contribute to a continued dilatation of cells till the cell membrane breaks down and leads to the liberation of cellular contents, which triggers an intense inflammatory response. By releasing inflammatory factors, pyroptosis can activate autoimmune cells. In recent years, an accumulating number of reports have illustrated the feasibility and curative potential of employing pyroptosis to participate in antitumor immunity via distinct targeting and delivery methods. Moreover, “cold tumor” corresponds to immune-silent, “hot tumor” corresponds to antitumor immune killing and further immune stimulation (Fan et al., 2019), “warming tumor” corresponds to induction of tumor cell pyroptosis (Gao et al., 2020), “warm tumor” corresponds to immune activation and infiltration (Erkes et al., 2020). Also, immune cells are a strong potential force in preventing or slowing tumor growth, which is related to tumor aggression and metastasis (Loveless et al., 2021). Meanwhile, there is growing evidence of direct or indirect interactions between pyroptosis and immune in the microenvironment of glioma, although the underlying mechanisms remain unclear.

Shao et al. (2021) developed a model of pyroptosis regulators as promising features and identified their association with clinical-pathological factors. Nevertheless, the majority of the proposed prognostic models for glioma contain only mRNA transcriptome profiles and are insufficient to achieve satisfactory performance. Within this research, we hypothesized that pyroptosis and immune interactions could provide prognostic value for patients with glioma. According to the genetic data of The Cancer Genome Atlas (TCGA) and the Chinese Glioma Genome Atlas (CGGA), TME invasion patterns were estimated in glioma patients, especially at the tumor stage, and then nine pyroptosis immune signatures were obtained from genetic or epigenetic features by analyzing multi-omics data. In our study, the pyroptosis immune prognostic model (PIPM) was developed based on pyroptosis immune signatures and the risk score based treatment strategy for glioma was developed. It is anticipated that the results of this study will provide a more complete genomic landscape of pyroptosis immune, which could provide better prognostic and therapeutic indicators for glioma.

## Materials and Methods

### Data Extraction

The normalized gene expression profile (FPKM) and clinical data of 698 glioma samples were collected from TCGA, and the RNA_seq and clinical data of 1018 glioma samples were from CGGA. A total of 598 profiles of the Illumina 450 k DNA methylation array were obtained from https://portal.gdc.cancer.gov. The mutation rate and copy number variation (CNV) frequency were gathered from cBioPortal. The tumor immune dysfunction and exclusion (TIDE) score was computed online (http://tide.dfci.harvard.edu/) and the 18-gene T-cell inflammatory marker (TIS) score was calculated as an average value of log2-scale normalized expression of the 18 signature genes (Ayers et al., 2017).

### Recognizing Pyroptosis Regulators

In a recent study, Shi et al. discovered that *CASP1* and *CASP4/5* can be specific for the cleavage of *GSDMD* and that the type of *GSDMD* cleavage is critical for pyroptosis (Shi et al., 2015). These results were eventually validated by He et al. (2015). Subsequently, Orning et al. identified that increased accumulation of *CASP8* was another efficient way to trigger *GSDMD* cleavage (Orning et al., 2018). From then on, numerous investigations have started to reveal the contribution of gasdermins in cells. *CASP3* and *GZMB* were found to cleave *GSDME*, thereby transforming apoptosis into pyroptosis (Rogers et al., 2017; Zhang et al., 2020). When *GSDMB* is split by *GZMA*, apoptosis can also be transformed into pyroptosis (Zhou et al., 2020). Therefore, we selected 11 genes (*CASP1*, *CASP3*, *CASP4*, *CASP5*, *CASP8*, *GSDMB*, *GSDMC*, *GSDMD*, *GSDME*, *GZMA*, and *GZMB*), which are strongly associated with pyroptosis and serve as pyroptosis regulators.

### Consensus Clustering

Consensual clustering uses the k-means approach to recognize unique pyroptosis patterns linked to the expression value of pyroptosis regulators. The number and stability of clusters were determined by the consensus clustering algorithm using the “ConsensuClusterPlus” package. We conducted 1,000 times iterations to ensure the robustness of our classification (Wilkerson and Hayes, 2010).

### Analyses of Mutation Subtypes

Somatic mutation and CNV profiles were gathered from the TCGA data portal. Somatic mutation data classified according to the mutation annotation format were performed by applying the R package “maftools” (Koboldt et al., 2012; Mayakonda et al., 2018).

### Identification of Pyroptosis Immune Subgroups and Relevant Prognostic Factor Differentially Expressed Genes

The pyroptosis and immune status were further incorporated into a two-dimension index, whereby patients were classified into four pyroptosis immune subgroups, i.e., “low-immunity and low-pyroptosis (LILP),” “low-immunity and high-pyroptosis (LIHP),” “high-immunity and high-pyroptosis (HIHP),” and “high-immunity and low-pyroptosis (HILP)” groups. The differentially expressed genes (DEGs) were gained by comparing the expression of the LILP and LIHP, LILP and HIHP, LILP and HILP, and HILP and HIHP groups (|log2FC|>1, FDR-adjusted *p* <0.05). Then, the intersection of the DEGs corresponding to the four pairs of groups was taken to obtain 55 hub genes. For DNA methylation, the R package “ChAMP” was employed to handle the Illumina Infinium 450 k DNA methylation array data. Deletions of more than 20% were screened and 598 samples were applied. The remaining missing values were statistically inferred using the ChAMP inference function. The beta values were standardized by applying peak-based calibration. Additionally, the differential methylation probes (DMPs) and regions were respectively recognized using the “limma” package. The pyroptosis immune DMPs were also achieved by the expression comparison between the LILP and LIHP, LILP and HIHP, LIHP and HILP, and HILP and HIHP groups (|beta|>0.3, FDR-adjusted *p* <0.01). Then, we obtained 24 DMPs by taking their intersection. The 24 DMPs (cg17601191, cg00270878, cg10884288, cg06706894, cg16824643, cg14337655, cg09509952, cg06933574, cg09187007, cg08003353, cg16786640, cg00782200, cg03300177, cg01969701, cg10504751, cg01135464, cg02666008, cg12565681, cg00467244, cg02793828, cg08442798, cg13172906, and cg08954277) correspond to 10 hub genes. Finally, we analyzed the mutation levels of the four pairs of groups of differential genes to obtain the intersection of one hub gene (*IDH1*). The three levels of hub genes, transcriptome level, methylation level, and mutation level, were summarized to obtain 66 genes, which we regard as pyroptosis immune genes. To acquire pyroptosis immune prognostic genes, univariate and multivariate cox regression were further applied among all 66 pyroptosis immune genes. Taken together, nine pyroptosis immune signatures were obtained. Those with a *p* <0.05 were considered significant.

### PIPM Predicts Effective Response to Postoperative Immunotherapy and Identifies the Impact on Pan-Cancer

PIPM was constructed by predicting regression for pyroptosis immune signatures, and we analyzed the survival, ROC, risk factor, stemness, and the response to immunotherapy of PIPM. Finally, we performed the correlation between the pan-cancer risk score and immune cells, stemness, and ESTIMATE score.

### Effect of Pyroptosis Immune Prognostic Model Risk Score in Mutations, Clinical Features and Stemness Index

The differences in tumor mutational burden load (TMB), tumor stemness indices (TSI), and the PIPM risk score in PIPM were evaluated using the Kruskal–Wallis test, and the correlation between the TSI, TMB, and PIPM risk score was evaluated by the Pearson correlation coefficient. Furthermore, the dedication to overall survival (OS) was calculated by the Kaplan–Meier algorithm. We also continued to conduct the relationship between the PIPM risk score and clinical characteristics.

### Significance of the Pyroptosis Immune Prognostic Model in Chemotherapeutic Sensitivity

To evaluate the clinical efficacy of PIPM for glioma treatment, the algorithm developed by Geeleheret et al. (Geeleher et al., 2014a) and the “pRRophetic” package (Geeleher et al., 2014b) were used to calculate the IC50 of commonly used chemotherapeutic agents in the TCGA project. The AJCC guidelines recommend 30 common antitumor drugs, like Adriamycin, Vinblastine, Cisplatin, and Imatinib for cancer treatment. The Wilcoxon test was used to compare the differences in IC50 of common antitumor drugs in high and low-risk groups, and the results are shown as box plots.

### Immune Infiltration

ssGSEA was used to describe the relative infiltration of 28 immune cells in the TME. Characteristic gene panels for each immune cell type were obtained from a recent paper (Jia et al., 2018).

### Statistical Analysis

All data were executed with R version 4.0.5 and its appropriate packages. ESTIMATE score was calculated by using the “estimate” package (Yoshihara et al., 2013). The lasso algorithm was performed by using the “glmnet” package (McEligot et al., 2020). Appropriate standard statistical tests were used to analyze the data. Adjustment for multiple testing was performed using the FDR approach. To ascertain independent risk factors for survival, univariate and multivariate cox regression was performed to correct for covariates.

## Result

### Landscape of Pyroptosis Regulators in Glioma

**FIGURE 1:**
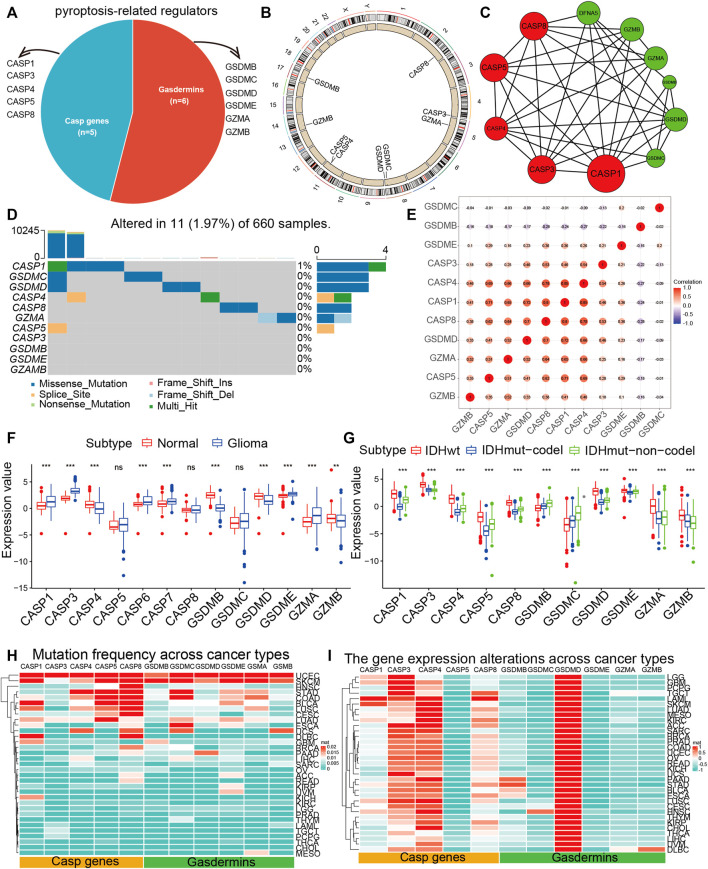
The landscape of pyroptosis regulators. **(A)** The proportion of gasdermins and CASP genes among pyroptosis regulators. **(B)** The position of CNV variation of pyroptosis regulators on 23 chromosomes from the TCGA-glioma cohort. **(C)** The PPI between pyroptosis regulators. The size of the circle indicated the strength of the connection of each node. **(D)** The mutation co-occurrence and exclusion analyses for 11 pyroptosis regulators. Co-occurrence, green; exclusion, purple. **(E)** Spearman correlation analysis of the pyroptosis regulators. **(F)** The expression of 11 pyroptosis regulators between normal and glioma samples. **(G)** The expression of 11 pyroptosis regulators between molecular subtypes. The top and bottom ends of the boxes indicated the quartile range of values. **(H)** The mutation frequency of pyroptosis regulators across 33 tumor types. **(I)** The gene expression of pyroptosis regulators across 33 tumor types.

The overview of our study was shown in Supplementary Figure S1. We retrospected pieces of literature and planned a catalog of 11 genes that served as pyroptosis regulators, including six gasdermins and five CASP genes, and found the variations of all pyroptosis regulators were common and mostly focused on copy number amplification in TCGA (Figure 1A, B). Then, we ascertained the alterations of the nine pyroptosis regulators featuring CNV on the chromosome. These results indicated that the CNV status of these nine regulators is relevant to the progression and occurrence of glioma. Analysis of the interactive patterns among the 11 pyroptosis regulators showed that *Caspase 1* (*CASP1*) was the pivotal node of the pyroptosis regulators, followed by *Caspase 3* (*CASP3*), *Caspase 5* (*CASP5*), and *Caspase 8* (*CASP8*), and its associations with *Caspase 4* (*CASP4*), *Gasdermin D* (*GSDMD*), *Granzyme A* (*GZMA*), and *Granzyme B* (*GZMB*) were also endorsed by the STRING database (Figure 1C). Among the 660 samples, the frequency of alteration in 11 pyroptosis regulators was 1.97% (11 mutations), which were mostly missense mutations. *CASP1* displayed the highest mutation frequency, followed by *Gasdermin C* (*GSDMC*), while *CASP3*, *Gasdermin B* (*GSDMB*), *Gasdermin E* (*GSDME*), and *GZAMB* did not display any mutations in glioma samples (Figure 1D). Subsequently, consequent investigations indicated a considerable association of co-occurrence mutation between *CASP5* and *CASP1*, *GSDMC*, and *GSDMD* (Supplementary Figure S2A). This high-incidence co-occurrence mutation indicates resistance to specific treatment targeting only one of the mutations, alternatively, indicates functional cooperative and, more importantly, the potency of combination therapy for glioma (Nissan et al., 2014). We further performed the correlation of the co-expression of regulators and discovered a considerable relevance between *CASP4* and other regulators, with the strongest correlation coefficient (0.85) between *CASP4* and *CASP1* (Figure 1E). Furthermore, we investigated the relevance between the expression patterns of these pyroptosis regulators and molecular characteristics. Of the 11 pyroptosis regulators, 8 revealed remarkable distinctions between glioma and normal tissues, while *CASP5*, *CASP8*, and *GSDMC* did not (Figure 1F). All regulators were clearly distinguished into groups classified according to *IDH1* molecular subtypes (Figure 1G).

### Genetic Alterations of Pyroptosis Regulators Across Cancer Types

Because of the above results, we identified 11 pyroptosis regulators that were significantly different expression and mutation levels in glioma and further explored its impact on pan-cancer. Cancer types with a higher overall mutation burden (such as UCEC, STAD, and SKCM) also displayed a higher mutation frequency in pyroptosis regulators. We discovered that *CASP1*, *CASP5*, *CASP8*, *GSDMC*, *GSDME*, and *GSMA* showed higher mutation frequencies (Figure 1H, Supplementary Table S1). Besides, we performed GO enrichment on 11 hub genes and found that they were primarily enriched in “pyroptosis,” “the execution phase of apoptosis,” and “response to tumor necrosis factor,” which further proved the correctness of our study on pyroptosis regulators (Supplementary Figure S2B). We further calculated the CNV alteration frequency for all pyroptosis regulators and revealed that CNV alterations were widespread. *GSDMB*, *GSDMC*, *GSDMD*, *GZMB*, and *GSDME* displayed extensive CNV amplification across cancer types (Supplementary Figure S2C, Supplementary Table S2). In contrast, *CASP1*, *CASP3*, *CASP4*, *CASP5*, *CASP8*, *GZMB*, and *GSDME* maintained prevalent CNV deletions (Supplementary Figure S2D, Supplementary Table S3). An attractive issue is whether alterations in these genes influence the expression of pyroptosis regulators. Therefore, we performed the perturbation expression of pyroptosis regulators across another nearly 10,000 samples, representing 33 cancer types, and observed *CASP3*, *CASP4*, *CASP8*, and *GSDMD* were highly expressed in cancer cells (Figure 1I, Supplementary Table S4). We assumed that alterations in CNV are likely to be one of the principal mechanisms leading to perturbations in the expression of pyroptosis regulators. The pyroptosis regulators with CNV amplification were significantly more highly expressed in cancer cells (e.g., *GSDMD*), whereas the regulators with CNV deletion were significantly less expressed (e.g., *CASP1*, *CASP3*, and *CASP4*). Particularly, we revealed that *GSDMD* displayed the highest expression in 33 cancer types. In conclusion, these findings implied that cross-talk among the pyroptosis regulators exerts a vital role in the development and progression of most cancers types, including glioma.

### Recognition Pyroptosis Pattern in Glioma

**FIGURE 2:**
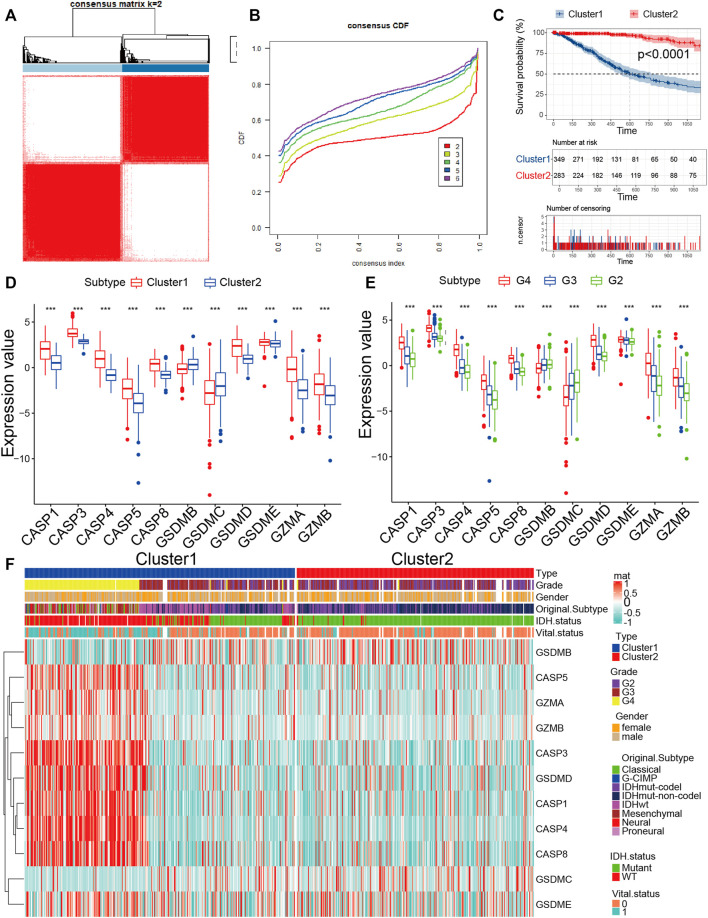
Identification of the pyroptosis pattern in glioma. **(A)** The consensus pyroptosis regulators matrix of gioma samples when k = 2 in TCGA cohorts. When the consistency of pyroptosis between two samples is high, they are more likely to be classified as the same cluster. **(B)** Consensus clustering cumulative distribution function (CDF) for k = 2–6 in TCGA cohort. **(C)** KM curves for the two clusters are based on 698 glioma samples from TCGA cohorts. **(D)** The expression of 11 pyroptosis regulators between high-expression pyroptosis and low-expression pyroptosis glioma samples. **(E)** The expression of 11 pyroptosis regulators among grades in glioma. **(F)** The expression profile of 11 pyroptosis regulators between the two clusters groups in the TCGA cohort. The heatmap columns depicted 698 glioma samples (*p < 0.05, **p < 0.01, ***p < 0.001, chi-square test).

Since 11 pyroptosis regulators had significant differences in tumor mutation, CNV, correlation, and internal expression profiles, we further explored the pyroptosis pattern. According to the expression levels of 11 pyroptosis regulators, we chose k = 2 for stable clustering of pyroptosis regulators based on their cumulative distribution functions and functional incremental regions. Afterwards, we obtained two distinct modulation patterns by applying the unsupervised clustering approach, consisting of 373 cases in pyroptosis-related cluster 1 and 325 cases in pyroptosis-related cluster 2 (Figure 2A, B, Supplementary Table S5). The survival advantage of cluster 2 was higher than that of cluster 1 (Figure 2C). We further performed heatmap analysis of the expression profile of the 11 pyroptosis regulators in glioma, and the results revealed that the expression level of the remaining nine genes, except for *GSDMB* and *GSDMC*, were significantly higher in cluster 1 than that in cluster 2. Moreover, we discovered that the expression values of all pyroptosis regulators were higher in patients with high-grade glioma (WHO III and IV), implying that the expression values of pyroptosis regulators could also reflect the tumor grade (Figure 2D–F). For *IDH1* mutation status, IDH1wt was mainly collected in cluster 1 and IDH1mut was mainly collected in cluster 2. For original subtypes, cluster 1 contained all eight subtypes, and cluster 2 mainly contained IDHmut-codel, IDHmut-non-codel, and IDHwt. These results not only imply the obvious survival difference between high and low pyroptosis subgroups but also indicate that the expression status of pyroptosis regulators was significantly correlated with the existing molecular subtypes of glioma. From the above findings, we found that cluster 1 is the high expression pyroptosis regulators group and cluster 2 is the low expression pyroptosis regulators group, which paved the way for our exploration of pyroptosis immune signatures. Moreover, the close correlation between our pyroptosis grouping and clinical traits further illustrated the accuracy and stability of pyroptosis patterns in glioma.

### Recognition Immune Pattern in Glioma

The interaction between pyroptosis and the immune system depends on complex cellular communication involving pyroptosis and immune cells (Legrand et al., 2019). Thus, we analyzed the immune status of glioma which was determined by the ssGSEA algorithm according to the immune infiltration in the tumor tissue, and glioma patients were classified into two groups by hierarchical clustering (Supplementary Figure S3A, Supplementary Table S6). Next, we explored the association between TME and immune groups. As illustrated in the results, we computed the distinction in immune infiltration between the two groups and observed that there was a significant enrichment of all types of infiltrating immune cells in one group, which we served as the high immunity group and the other as the low immunity group (Supplementary Figure S3C). Patients in the high immunity group had a superior survival than those in the low immunity group (Supplementary Figure S3B). The distribution of immune scores and stromal scores was considerably higher in the high immunity group than that in the low immunity group, and the reverse result for tumor purity (Supplementary Figure S3F). ICPs are essential for cancer immunotherapy with numerous ICPs activator and antagonists being evaluated in clinical oncology (Hodges et al., 2017; Thorsson et al., 2018; Galluzzi et al., 2020). We further analyzed their expression levels in distinct subtypes, forty ICPs genes were detectable in the TCGA cohort. For example, *CD70*, *CD86*, *CD200R1*, *CD40LG*, *CD244*, *CD40*, *CD48*, *CD274*, *CD80*, *CD27*, *CTLA4*, *IDO1*, *HAVCR2*, *ICOS*, *ICOSLG*, *LGALS9*, *LAG3*, *LAIR1*, *PDCD1*, *PDCDILG2*, *TNFRSF15*, *TNFRSF4*, *TNFRSF14*, *TNFRSF18*, and *TNFRSF9* were overexpressed in high immunity group in the TCGA cohort (Supplementary Figure S3E). Next, we calculated the association between HLA genes sets and two immunity types. As displayed in the results, the samples in the low immunity group have remarkably higher expression than the samples in the high immunity group (Supplementary Figure S3D). The observed distinction in the modulation of ICPs might have implications for combination immune therapies, and the variety of mechanisms that play a role in evoking them further emphasizes their biological significance.

### Recognition Pyroptosis Immune Subgroups in Glioma

**FIGURE 3:**
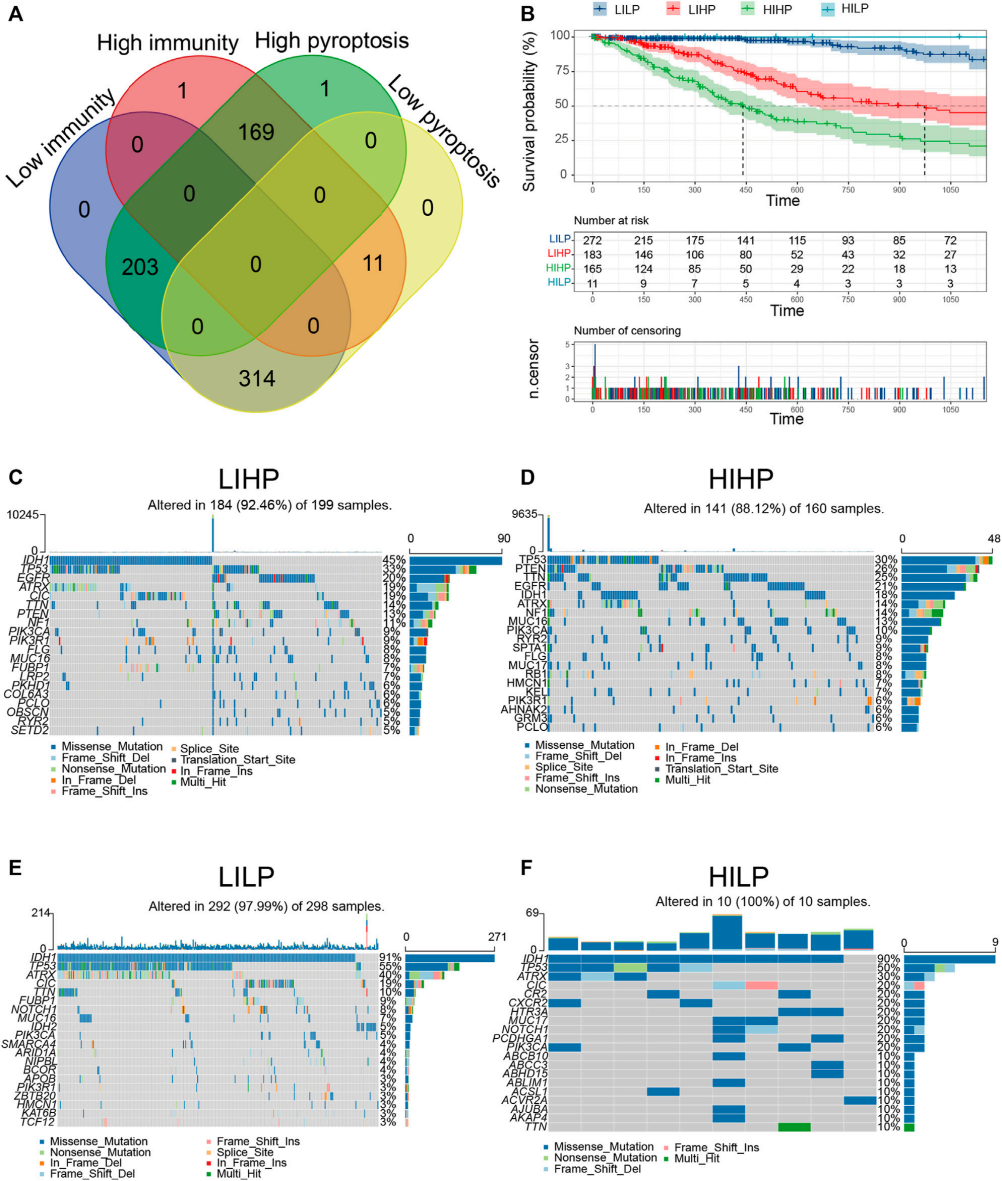
Identification of pyroptosis immune groups. **(A)** The overlapping patients were identified among four pyroptosis immune groups. **(B)** KM curves for patients in four pyroptosis immune groups. **(C–F)** The waterfall diagram displays the distribution of the top 20 most frequently mutation genes.

Based on the aforementioned pyroptosis and immune status, we further combined them into a two-dimension index, whereby patients were classified into four groups: “LILP,” “LIHP,” “HIHP,” and “HILP” groups (Figure 3A). Survival analysis indicated considerable distinction among the four groups (*p* <0.001), patients in the “HILP” group had the best survival, whereas those in the “HIHP” group had the worst prognosis (Figure 3B, Supplementary Table S7). This result echoes the previous analysis, and this stratification facilitates the following exploration of their underlying mechanistic differences.

### Mutation Landscape of Pyroptosis Immune Pattern

Relevant literature implies that the immune status is also likely to be in contact with mutation and that greater TMB and somatic mutation rates are associated with greater anticancer immunity (Rooney et al., 2015; Cao et al., 2019). Hence, we computed mutations and TMB for each patient applying the mutation dataset and analyzed them across all pyroptosis immune patterns. Among the four pyroptosis immune subtypes, LILP and HILP had the highest mutation rate (97.99 and 100%), followed by LIHP (92.46%), and HIHP (88.12%). The IDH1mut rate was the highest in LILP (91%) and HILP (90%), followed by LIHP (45%) and HIHP (18%, Figure 3C–F). IDH1mut considerably influences the outcome of glioma patients. Therefore, the distinction in IDH1mut among the pyroptosis immune subtypes is likely to be a factor influencing the survival of patients (Yan et al., 2009). Furthermore, we investigated the co-occurrence landscape by applying the top first 25 mutated genes with the comet algorithm. Ten pairs cases (*IDH1*-*PTEN*, *IDH1*-*EGFR*, *TP53*-*CIC*, *CIC*-*ATRX*, *FUBP1*-*CIC*, *FUBP1*-*TP53*, *IDH1*-*IDH2*, *PIK3CA*-*TP53*, *ZBTB20*-*TP53*, and *IDH1*-*PTEN*) displayed mutually exclusive mutations compared to the pervasive mutually exclusive landscape, indicating that they may have redundant effects in the common pathway and a selective advantage of retaining a copy of the mutation between them (Supplementary Figure S4A, B). The top 10 most significantly differentially mutated genes in the corresponding cohorts were illustrated in Supplementary Figure S4C–F. Interestingly, *IDH1* occupied the top one positions in four cohorts, which modulated diverse tumor-associated biological processes in glioma. TMB in HIHP and LIHP were significantly higher than that in LILP and HILP (Supplementary Figure S5A). After examining transcriptional alterations in the four aforementioned pyroptosis immune subgroups, it was further investigated whether genomic-level differences existed between the four subgroups. Somatic mutations, encompassing single nucleotide variants, single nucleotide polymorphisms (SNP), insertions, and deletions, were calculated and displayed using the “maftools” package. The SNPs and Total in the HIHP and LIHP were also higher than those in HILP and LIHP, while the most of genomic variants were missense mutations (60%) in the four pyroptosis immune subtypes (Supplementary Figure S5B, C). Hence, it is imperative to quantify the mutational types and reveal their potential significance. From the above results, we found that the four pyroptosis immune subtypes had significant survival and mutational differences, which provided a basis for further exploration of pyroptosis immune signatures.

### Identification Pyroptosis Immune Signatures

**FIGURE 4:**
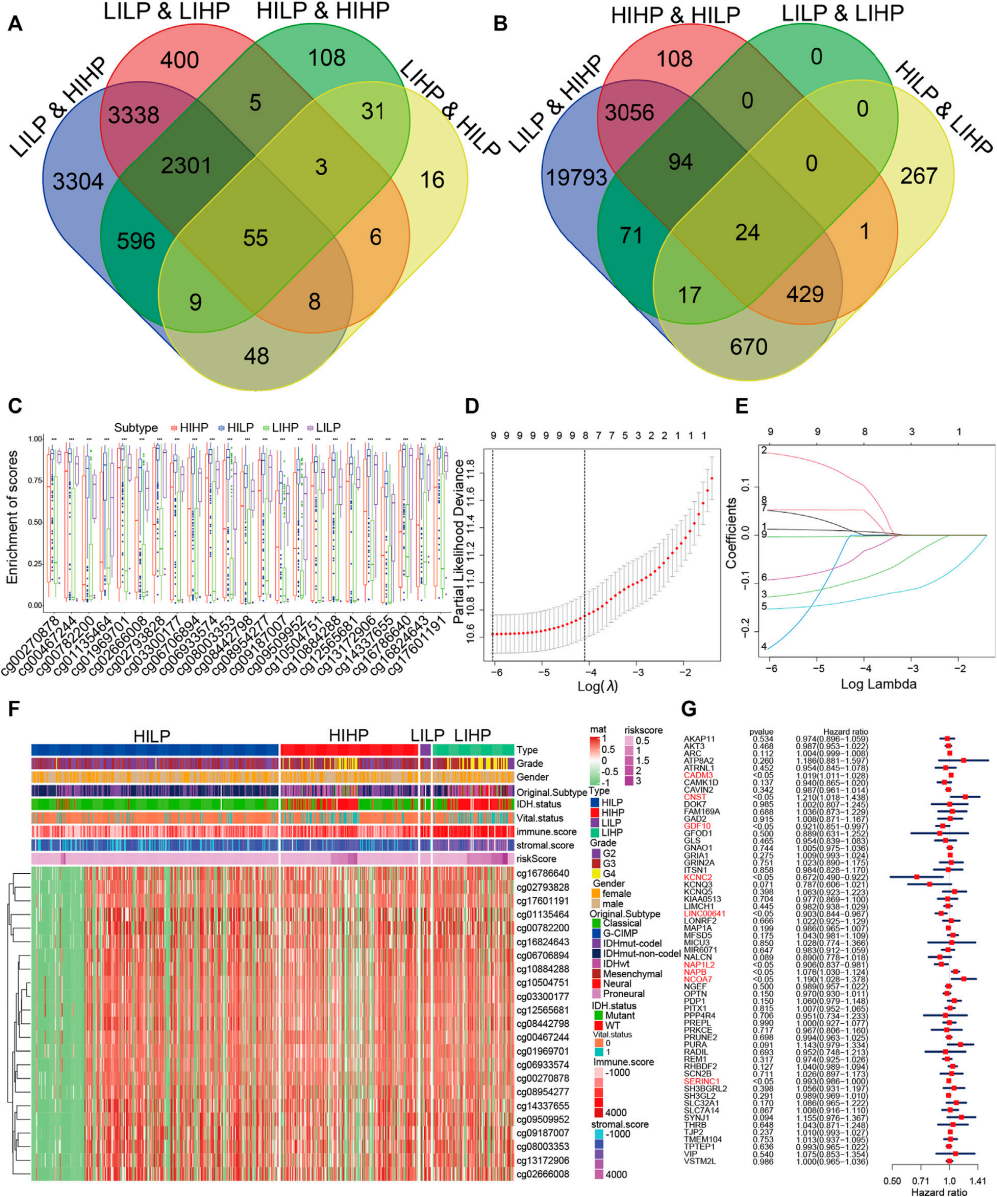
Identification of pyroptosis immune signatures. **(A)** The overlapped DEGs were identified among four pyroptosis immune groups. **(B)** The overlapped DMPs were identified among four pyroptosis immune groups. **(C)** The distribution of the 24 DMPs in the four pyroptosis immune groups. **(D)** Regression coefficient profiles of identified pyroptosis immune regulators in the TCGA cohort. **(E)** Ten-time cross-validation for tuning parameter selection in the TCGA cohort. **(F)** The relationship between the distribution of the 24 DMPs in the four pyroptosis groups with clinical traits. **(G)** Multivariate cox analyses of the association among pyroptosis immune signatures.

Due to distinct survival differences among the four pyroptosis immune subgroups, we performed differential gene analysis for the subgroups with the largest survival difference. Then we calculated the DEGs between subgroups HILP and HIHP, HILP and LIHP, LHLP and HIHP, LILP and LIHP (Supplementary Figure S6A–D). We used the Venn Diagram web tools and obtained 55 DEGs among the DEGs based on LILP and HIHP groups, the DEGs based on HIHP and HILP groups, and the DEGs based on LILP and LIHP groups, and the DEGs based on HILP and LIHP groups (Figure 4A). These DEGs were *GDF10*, *TMEM104*, *PDP1*, *ITSN1*, *LINC00641*, *GFOD1*, *PURA*, *VIP*, *KCNC2*, *ATRNL1*, *SH3GL2*, *NCOA7*, *SLC7A14*, *COX20P1*, *PRKCE*, *MIR6071*, *PPP4R4*, *NAPB*, *SH3BGRL2*, *MAP1A*, *NGEF*, *GLS*, *ATP8A2*, *MICU3*, *VSTM2L*, *FAM169A*, *NALCN*, *CADM3*, *LIMCH1*, *MFSD5*, *NAP1L2*, *THRB*, *KCNQ5*, *LONRF2*, *GRIN2A*, *CAVIN2*, *KIAA0513*, *KCNQ3*, *AKT3*, *SLC32A1*, *PRUNE2*, *CAMK1D*, *SYNJ1*, *SCN2B*, *GRIA1*, *GAD2*, *PREPL*, *AKAP11*, *IDH2*, *TPTEP1*, *OPTN*, *TJP2*, *SERINC1*, *REM1*, and *CNST*. The inability to sustain normal DNA methylation, including hypermethylation in CpG islands and hypomethylation in CpG-poor regions, enhances the sensitivity to induce tumor formation and progression (Koch et al., 2018; Soozangar et al., 2018). Hence, we intended to inspect and contrast the impact of DNA methylation patterns in pyroptosis immune subgroups. We also applied the Venn Diagram and obtained 24 DMPs among the DMPs based on LILP and HIHP groups, the DMPs based on HIHP and HILP groups, the DMPs based on LILP and LIHP groups, and the DMPs based on HILP and LIHP groups (Figure 4B). We further examined the distribution of the 24 DMPs in the four pyroptosis immune subgroups and the relationship between the 24 DMPs and clinical traits. The results displayed that the expression of 24 DMPs in the HIHP and LIHP groups was obviously lower than that of HILP and LILP, contrary to the results of the higher immune score and stromal score (Figure 4C). Moreover, we found that patients with G4 grade tumors and IDHwt were predominant in the low expression group of 24 DMPs, and the low expression of 24 DMPs often corresponded to the high expression of genes, so their corresponding genes also might be the pyroptosis immune genes affecting the prognosis of glioma (Figure 4F). Simultaneously, the 24 DMPs correspond to 10 genes, which were *ARC*, *C19orf35*, *DOK7*, *GNAO1*, *GNAO1*, *MEGF6*, *PITX1*, *RADIL*, *RHBDF2*, and *SLC22A11*. Finally, we combined the 55 DEGs and 10 genes, and 1 mutation DEG for a total of 66 pyroptosis immune signatures. To further obtain more accurate pyroptosis immune prognosis signatures, we performed univariate and multivariate cox regressions from 66 signatures and obtained 9 pyroptosis immune signatures. The nine signatures were *CADM3*, *CNST*, *GDF10*, *KCNC2*, *LINC00641*, *NAP1L2*, *NAPB*, *NCOA7*, and *SERINC1*.

### Construction Pyroptosis Immune Prognostic Model and Validation of the Pyroptosis Immune Prognostic Model Risk Score in the Cancer Genome Atlas Cohort

To verify the importance of these nine pyroptosis immune signatures for the survival of glioma patients, we constructed PIPM by lasso regression for nine pyroptosis immune signatures (Figure 4D, G, Supplementary Table S8). Then, we performed a correlation between pyroptosis regulators and pyroptosis immune signatures. The results revealed that the pyroptosis immune signatures were significantly and negatively correlated with the pyroptosis regulators (Supplementary Figure S7B). Furthermore, we constructed the scoring system based on PIPM, and the samples were classified into high and low-risk cohorts based on median-PIPM risk score. The Kaplan–Meier curve showed that overall survival (OS) was poorer in the high-risk cohort compared to the low-risk cohort (*p* <0.0001, Supplementary Figure S8A). The survival advantage was better in the low-risk group than that in the high-risk group, regardless of the treatment (Supplementary Figure S8C, D, Supplementary Table S9). The distribution of the PIPM risk score and OS were verified in the risk curve, and the results showed that the expression of nine signatures was significantly negatively correlated with PIPM risk score and OS (Supplementary Figure S8B). The time ROC showed the average AUC values of 1-, 3-, 5-, 7-, and 10-year prognosis predictions on the TCGA cohort reached 0.86, 0.91, 0.89, 0.85, and 0.8 (Supplementary Figure S8E). The survival ROC which showed the average AUC values of the PIPM risk scores for the 1-, 3-, and 5-year prognostic predictions reached 0.852, 0.900, and 0.869 and were significantly higher than for other clinical traits (Supplementary Figure S8F–H). These results illustrated the accuracy of PIPM in predicting the prognosis of patients with glioma. Then, we ranked the glioma samples according to their risk score (from low to high risk score) and tested whether any demographic/molecular/clinical feature was related to PIPM (Supplementary Figure S9A). The results showed that the PIPM risk score gradually increased with increasing grade, with IDH1mut patients having lower PIPM risk score than IDH1wt patients and 1p19q non-codel patients having significantly higher PIPM risk score than 1p19q codel patients (Supplementary Figure S9B–D). The distribution of diverse tumor grades within the two subgroups revealed that G4 was exclusively presented in the high-risk group (Supplementary Figure S9E). The distribution of IDH1mut within the two subgroups showed that IDH1mut accounts for the majority of the low-risk group (Supplementary Figure S9G). The distribution of 1p19q codel was obviously higher in the low-risk than in the high-risk group (Supplementary Figure S9F). These findings validate the accuracy of PIPM for predicting clinical and molecular features. Further analysis of this scoring system could help us understand the deeper relationship between pyroptosis and the immune, but further validation is required.

### Validation of the Pyroptosis Immune Prognostic Model Risk Score in the Chinese Glioma Genome Atlas Cohort

To further confirm the accuracy of the PIPM in predicting patient prognosis, we also validated PIPM in the CGGA cohort, and the Kaplan–Meier survival analysis showed the same results as the TCGA cohort (Supplementary Figure S10A). The survival advantage was higher in the low-risk group than that in the high-risk group, regardless of the treatment (Supplementary Figure S10E, F, Supplementary Table S10). The time ROC showed the average AUC values of 1-, 3-, 5-, 7-, and 10-year prognosis predictions on the CGGA cohort reached 0.71, 0.76, 0.77, 0.77, and 0.82 (Supplementary Figure S10H). We identified a quantitative analysis of the relationship between the PIPM risk score and the IDH1mut types, recurrence status, and 1p19q codel in the CGGA cohort. These findings are consistent with those in the TCGA cohort (Supplementary Figure S10B–D). The distribution of recurrence status within the two subgroups showed that recurrent and secondary status accounts for the majority of the high-risk group (Supplementary Figure S10I). The distribution of the IDH1mut within the two subgroups showed that IDH1 mutations account for the majority of the low-risk group (Supplementary Figure S10K). The percentage of 1p19q codel was significantly higher in the low-risk group than in the high-risk group (Supplementary Figure S10G). Taken together, these findings further demonstrate the accuracy of PIPM in predicting patient prognosis and molecular typing predictions.

### Connectivity Map Analysis Determines Potential Compounds/Inhibitors Targeting the Pyroptosis Immune Prognostic Model

We performed a differential analysis for the high and low risk groups. GO, KEGG, and GSEA were conducted to predict the potential performance of DEGs between the high- and low-risk groups, and as expected, the DEGs were enriched in pyroptosis and immune pathways, such as neutrophil degranulation, neutrophil activation involved in immune response, pathways of neurodegeneration-multiple diseases, hallmark apoptosis, hallmark DNArepair, and hallmark complement (Supplementary Figure S11A–C). We applied the Connectivity Map (CMap), a data-driven, systematic method to discover associations between genes, chemicals, and biological conditions, to find candidates that may target pathways related to compounds that are related to pyroptosis immune. We found the degree of enrichment of 11 kinds of compounds associated with pyroptosis immune (Supplementary Figure S11D). CMap mode-of-action (MoA) analysis of the eight compounds indicated eight mechanisms of action shared by the above compounds (Supplementary Figure S11E). We observed that alprenolol shared MoA as an Adrenergic receptor antagonist, econazole shared MoA as a bacterial cell wall synthesis inhibitor, Lanosterol demethylase inhibitor, and Sterol demethylase inhibitor. Benzbromarone shared MoA as a chloride channel blocker and terguride shared MoA as a dopamine receptor agonist and serotonin receptor antagonist. We observed the mechanism of action corresponding to different compounds to provide options for targeted therapy of glioma.

### Relationship Between Tumor Mutational Burden Load, T-Cell Inflammatory Markers, Tumor Stemness Indices, and Pyroptosis Immune Prognostic Model Risk Score

The biomarkers, such as TIDE and TIS, have been reported to predict patient response to immunotherapy (Chen et al., 2021). Higher TIDE prediction scores are associated not only with poorer immune checkpoint inhibition treatment outcomes but also with poorer survival under antiPD1 and antiCTLA4 therapy. High TIS scores also are associated with longer survival (Jiang et al., 2018). Patients with high TMB levels who were treated with nivolumab had significantly better tumor remission and survival benefits than chemotherapy (Hellmann et al., 2019). We revealed that the high-risk group showed higher TMB, TIS, lower TIDE, MSI Expr Sig, dysfunction, exclusion, and poorer prognoses than that of low-risk group, this result showed high risk group may be a better response to ICB therapy than that of low risk group (Supplementary Figure S12A–C, Supplementary Table S10). The distribution of the aforementioned three indicators further identified the advantages of PIPM in predicting the efficacy of immunotherapy in patients. We then investigated the correlation of TMB and TIS with risk socre, and the results showed a high correlation between them, 0.49 and 0.58, respectively (Supplementary Figure S12D, E, Supplementary Table S11, S12). Stemness differences appeared between the groups of the PIPM; specifically, the dedifferentiation phenotype was pronounced in the high-risk group, while the differentiation phenotype was pronounced in the low risk group (Supplementary Figure S13A, B, Supplementary Table S13). Moreover, mDNAsi, DMPsi, EREG-mDNAsi, ENHsi, and EREG-mRNAsi were actively and significantly correlated with the PIPM risk score, and the correlations were significantly 0.58, 0.54, 0.58, 0.57, 0.23, and 0.3 respectively (Supplementary Figure S13C–H). In conclusion, there was significant distinction in the degree of tumor differentiation, TMB, TIDE, and TIS between the two groups of PIPM. We conducted a comprehensive analysis of PIPM using three different marks (TMB, TIDE, and TIS) that evaluated the response to ICB treatment, which further supports the accuracy and robustness of PIPM.

### Immune Landscape of the Pyroptosis Immune Prognostic Model

To assess the immune status of PIPM, ssGSEA was used to conduct the immune infiltration of glioma samples. The immune cell component in different subgroups displayed similar immune cell scores in the TCGA and CGGA cohorts (Supplementary Figures S14A, S15A). Furthermore, we computed the distinction in immune cell infiltration between the two groups and discovered that 28 kinds of immune cells were considerably abundant in the high-risk group (Supplementary Figures S7C, S15B). Furthermore, we also found that forty ICPs were detectable in the TCGA cohort. For example, *BTLA*, *CD200*, *CD244*, *CD27*, *CD274*, *CD28*, *CD200R1*, *CD70*, *CD40*, *CD40LG*, *CD48*, *CD80*, *CD86*, *CTLA4*, *LAG3*, *PDCDILG2*, *HAVCR2*, *IDO1*, *ICOS*, *LGALS9*, *ICOSLG*, *PDCD1*, *TMIGD2*, *TNFRSF14*, *LAIR1*, *TNFRSF18*, *TNFRSF25*, *TNFRSF4*, *TNFRSF8*, *TNFRSF9*, *TNFRSF15*, and *TNFSF4* were upregulated in the high risk group in the TCGA cohort (Supplementary Figure S15D). Then, Pearson correlation coefficients between PIPM risk scores and ICPs were calculated. There was a positive relevance between PIPM risk scores and the listed thirty-eight ICPs, except for ADORA2A and CD200 (Supplementary Figure S7A). In addition, the upregulated ICPs in the TCGA and CGGA cohorts were also concentrated in the high-risk group (Supplementary Figure S12B). Moreover, 23 ICD genes were detected in the TCGA cohort, 22 of which (95.6%) were remarkably distinct between the two subgroups. For instance, *ANXA1*, *CALR*, *CCL2*, *CCR2*, *CGAS*, *CXCL1*, *HMGB1*, *CXCL10*, *FPR1*, *CXCR2*, *CXCR3*, *P2RY2*, *TLR2*, *TLR3*, *TLR9,* and *ZBP1* were considerably increased in the high-risk group in the TCGA cohort (Supplementary Figure S14E). In addition, the upregulated ICDs in the CGGA cohort were also the same result as the TCGA cohort (Supplementary Figure S15C). To verify the immune reliability of PIPM, we next performed the linkage between two subgroups and the six pan-tumor immune subtypes (C1–C6) previously reported (Thorsson et al., 2018), and the percentage of C4 and C6 was considerably higher in the high risk group than in the low-risk group. The high percentage of C4 and C6 samples in the high-risk group was in line with the worst prognosis in six clusters, as shown in Supplementary Figure S14C. Meanwhile, we observed the proportion of IDH1mut status in two subgroups, and the results showed that IDH1mut was predominant in the low risk group, which was consistent with a better prognosis in the low risk group (Supplementary Figure S14D). The robustness of the PIPM was strengthened by the relative similarity of immune cells content for the two cohorts (TCGA and CGGA).

### Comparison of the Sensitivity to Antitumor Drugs Among Patients With Distinct Pyroptosis Immune Prognostic Model Risk Score

**FIGURE 5:**
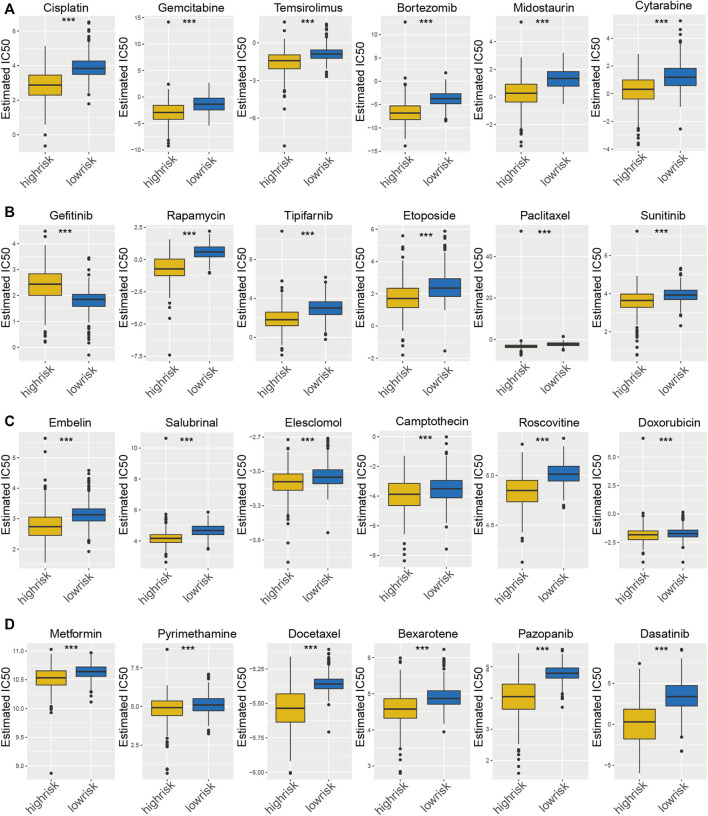
Estimated drug sensitivity in patients with high and low PIPM risk. **(A–D)** The chemotherapeutic reaction of PIPM for 30 prevalent chemotherapy drugs.

Distinct glioma subgroups in PIPM should guide clinical treatment. Thus, we compared the sensitivity of the high-risk and low-risk groups to 30 common anticancer drugs to select potential glioma treatment modalities. A total of 24 chemotherapeutic agents had significantly different IC50 estimates between the high-risk and low-risk groups (Figure 5A–D). Patients in the low-risk subgroup may be sensitive to these drugs. Under these circumstances, these drugs could be applied for the therapy of glioma in the future.

### Role of Pyroptosis Immune Prognostic Model Risk Score in Immunotherapy

Immune checkpoint blocking strategies, such as PD-1 and PD-L1, are hopeful therapeutic approaches that allow patients to achieve survival benefits. Considering that some patients are tolerant to immunotherapy, there is a need to identify biomarkers that are sensitive to immunotherapy. In this research, the PIPM risk score was utilized to evaluate the response to immunotherapy. We paired comparative analyses of the expression patterns of pyroptosis immune regulators in the GSE121810 cohort. To thoroughly analyze the relationship between the PIPM risk score and immunotherapy, we performed a further analysis of patients receiving pembrolizumab (a monoclonal antibody against PD-1) (Ganesh et al., 2019). We observed that the PIPM risk score was considerably lower in the group with the response (CR and PR) than in the group with disease (PD, Supplementary Figure S16H, Supplementary Table S14). Furthermore, we discovered that the proportion of PD was prominently higher in the high PIPM risk score group than in the low PIPM risk score group (Supplementary Figure S16E). In addition, the obvious survival benefit of the low PIPM risk score group was discovered by survival analyses, indicating that the PIPM risk score can represent sensitivity to immunotherapy (Supplementary Figure S16F). Finally, we performed the correlation analysis of PIPM risk score with TMB and Neotigen, respectively, and the results showed a high correlation between them (Supplementary Figure S16G).

Atezolizumab, a monoclonal antibody targeting PD-L1, has been endorsed by the FDA for tumor therapy (Zettler and Nabhan, 2018). We computed the PIPM risk score of samples from patients who received atezolizumab and detected a distinct survival benefit in the low PIPM risk score group (Supplementary Figure S16A). Furthermore, we performed the distribution of the PIPM risk score from patients with distinct responses to immunotherapy and showed that the PIPM risk score in the CR/PR group were considerably lower than those in the SD/PD group, indicating that the PIPM risk score can represent the sensitivity of patients to immunotherapy (Supplementary Figure S16B, Supplementary Table S15). Moreover, the percentage of distinct tumor stages and grades within the two subgroups revealed that III and IV grades are majorly present in the high-risk group (Supplementary Figure S16C). Finally, we observed that the proportion of SD/PD was considerably higher in the high PIPM risk score group than in the low PIPM risk score group (Supplementary Figure S16D).

Taken together, our study firmly revealed that the PIPM risk score was substantially associated with response to antiPD-1/L1 immunotherapy. Our PIPM risk score system may contribute to clinicians determining patients with sensitivity to ICB therapy, and recognizing patients who are more appropriate for immunotherapy.

### Efficacy of the Pyroptosis Immune Prognostic Model Risk Score Across Tumor Types

**FIGURE 6:**
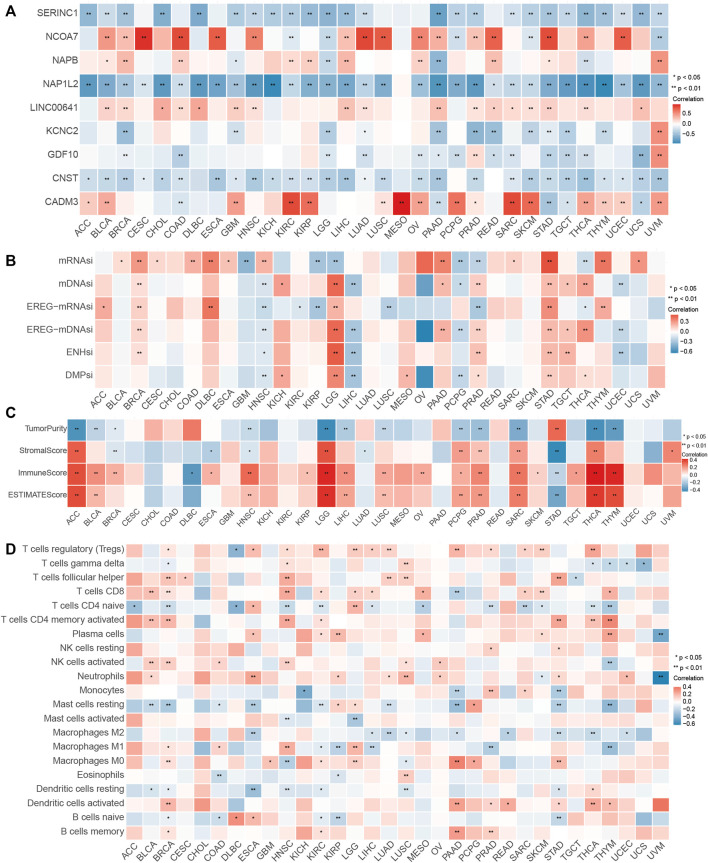
Performance of the PIPM risk score across tumor types. **(A)** Association between the PIPM risks core and immune cells for each cancer type. **(B)** Association between the PIPM risk score and stemness indices for each cancer type. **(C)** Correlations between the PIPM risk score and ESTIMATE score for each cancer type. **(D)** Correlations between the PIPM risk score and pyroptosis immune signatures for each cancer type.

Given the robust relationship between the PIPM risk score and immunotherapy response described earlier, we further investigated the efficacy of the PIPM scoring system across cancer types and discovered a considerable correlation between the expression level of pyroptosis immune regulators and the PIPM risk score in each cancer type. The expression values of these regulators, UVM, UCS, THCA, PRAD, PAAD, LIHC, and BRAC, were positively correlated with the PIPM risk score, while the others were negatively correlated (Figure 6A, Supplementary Table S16). Furthermore, we illustrated the expression of pyroptosis immune regulators across another∼10000 samples and revealed that *CNST*, *GDF10*, *KCNC2*, *NAP1L2*, *NCOA7,* and *LINC00641* also revealed higher expression in cancer cells (Supplementary Figure S17C). As shown in Supplementary Figures S17A,B, and Supplementary Table S17, we observed that the PIPM risk score was also an important prognostic (in terms of PFI or OS) risk factor for most cancers. The association between the PIPM risk score and the adverse prognosis of all tumor types, encompassing glioma, may indicate the origin of cancer cells or the impact of pyroptosis immune regulators. For instance, the PIPM risk score had the higher HR for the prognosis of PAAD, concordant with the high malignancy of pancreatic cancer. Then, we observed the significant association between the Estimate score and the PIPM risk score in addition to CESC, CHOL, COAD, KICH, KIRC, MESO, READ, UCEC, and UCS (Figure 6B, Supplementary Table S18). The proportion of 22 kinds of immune cells was identified as immune infiltration of tumor immune microenvironment (TIME). We calculated the association between the proportion of 22 immune cells and the PIPM risk score and discovered different trends in the association for 32 cancer types, except CHOL. The proportion of regulatory T cells CD4 Memory, T cells CD4 naïve, and Macrophages M1 was relevant to the PIPM risk score of most cancer types (Figure 6C, Supplementary Table S19). Notably, these cells were of the antitumor type, which to some extent, suggest that the pyroptosis immune regulators promote antitumor immunity. Finally, we found an association between the PIPM risk score and stem cell indices for 32 cancers except for CHOL, LUAD, SKCM, and UVM (Figure 6D, Supplementary Table S20). Such cancers with high mDNAsi and a reduced leukocyte fraction were anticipated to be less susceptible to immune checkpoint therapy. We comprehensively evaluated the relevance of the PIPM scoring system to the pan-cancer field and deeply explored the excellent features of the PIPM risk score in pan-cancer, laying the foundation for the widespread application of PIPM in oncology.

## Discussion

In contrast to apoptosis, which is essentially an immune-tolerant process, pyroptosis has a molecular mechanism that triggers a strong inflammatory response and in some cases is considered an ICD form (Zhang et al., 2020). Although the relationships between pyroptosis and anticancer immunity are not well understood, an increasing number of researches suggest that pyroptosis mediates tumor clearance by expanding immune activation and function (Wu et al., 2021). Furthermore, besides being automatically initiated by distinct stressors and an apoptotic-pyroptosis switch, tumor cell pyroptosis can be directly mediated by certain immune cells. Recent studies showed that pyroptosis is associated with anticancer immunity based on the involvement of *GSDM* proteins (Loveless et al., 2021). For instance, the expression of *GSDMD* and *GSDME* was linked to the activation of abundant immune cells in some solid tumors, such as dendritic cells, CD8+ T cells, NK cells, monocytes, neutrophils, CD4+ T cells, and macrophages. The expression of *GSDMD* and *GSDME* was also linked to immune factors, including IL-18, IFN-g, IL-1α, and IL-6. It indicates that pyroptosis can facilitate the immune system by activating immune cells and immune factors and may be involved in a positive feedback loop of antitumor immunity.

Given the highly variable prognosis of glioma, it is essential to develop a powerful classifier to stratify patients with distinct risks and outcomes, which is crucial to achieving maximum benefit from individualized treatment and timely follow-up. Thus, enormous endeavor and attention have been devoted to exploring the complicated mechanism of glioma, but the present knowledge, particularly regarding TME, therapeutic target, and prognostic aspects, is still far from satisfied. In the current research, we performed profiling of transcriptional profiles, somatic mutations, and methylation features to construct a tool that will help overcome this significant clinical problem. Our study provides a multi-dimensional analysis with multi-omics, multi-cohorts, and deep machine learning to assist us in understanding the impact of pyroptosis and the immune in glioma.

Pyroptosis regulators have rarely been studied in the previous paper (Shao et al., 2021). Now, pyroptosis immune regulators identified in this study could present underlying targets for the laboratory experimental design to elucidate molecular mechanisms in glioma. Furthermore, based on detected genetic or epigenetic alterations, we constructed PIPM-based pyroptosis immune signatures and found it to have preferable performance in predicting prognosis. Surprisingly, PIPM showed the highest accuracy of 3-year survival prediction than other years in the TCGA cohort. Meanwhile, promising results were also obtained in CGGA cohort.

More importantly, it has been published that pyroptosis promotes and sustains tumor cell death via activating the immune effector cells and augmenting the activity of immune cells (Wang et al., 2020). The pyroptosis and immune microenvironment play a crucial role in the suppressing development of cancers (Zhang et al., 2020). We discovered that the pyroptosis immune DEGs, mainly containing transcriptome level, mutation level, and methylation level, can more comprehensively identify pyroptosis immune targets. In our study, we observed a synergetic effect of pyroptosis and immune status on the prognosis of glioma patients. Higher pyroptosis status was related to poor prognosis, while higher immune status could reveal a better result. Therefore, it is worthwhile to further investigate the application of pyroptosis and immune status in glioma.

Due to the complexity of pyroptosis and immune activity in the TME, there are no published biomarkers that use multi-omics data to assess the pyroptosis and immune status. As tumors form regions of pyroptosis, tumor cells may receive suppression with the increased immune response (Fu, 2020). Moreover, it is not powerful to use a single transcriptomic to determine the pyroptosis status. We performed consensus clustering and hierarchical clustering on 698 glioma samples to obtain four pyroptosis immune groups. Then, the 66 pyroptosis immune genes were obtained based on multi-omics data of four pyroptosis immune subgroups. Finally, univariate and multivariate Cox regressions were performed on 66 pyroptosis immune genes to obtain 9 pyroptosis immune signatures. Important roles of the nine pyroptosis immune signatures have been described previously in several types of cancers. *CADM3*, also referred to as synaptic cell adhesion molecule 3 (*SynCAM3*), is part of the nectin family. *CADM3* includes two Ig-like C2-type (immunoglobulin-like) domains and one Ig-like V-type (immunoglobulin-like) structural region. The expression of isoform 1 is primarily in the adult and fetal brain, while the expression of *synCAM2* is high in the adult brain and weak in the placenta. Interaction of *CADM3*/*SynCAM3* with *EPB41L1* may modulate the structure or function of cell-cell junctions (Dong et al., 2006). *CNST* is an overall membrane-bound protein that is a binding partner for connexins, serves as a building block for gap junctions, and participates as a trans-Golgi network (*TGN*) receptor for connexin targeting to the plasma membrane and recovery from the cell surface (del Castillo et al., 2010). *GDF10* is a member of the BMP family and the TGF-beta superfamily. *GDF10* is distributed in the brain, femur, lung, skeletal, muscle, pancreas, and testis, and plays a major role in head formation and possibly multiple roles in skeletal morphogenesis (Zhao et al., 1999). *KCNC2* was a protein-coding gene. Disorders related to *KCNC2* contain extratemporal epilepsy and spinocerebellar ataxia 13. Its relevant pathways include aquaporin-mediated transport and potassium channels. GO annotations related to this gene include ion channel activity and deferred rectifier potassium channel activity (Sárvári et al., 2017). *LINC00641*, as a tumor suppressor, offers new potential therapeutic targets for glioma patients by targeting the miR-4262/NRGN axis in glioma (Yang et al., 2020). *NAP1L2* protein has a critical impact in modulating transcription in evolving neurons through the regulation of histone acetylation. The neuronal nucleosome assembling proteins regulate cell-type-specific processes of establishment/modification of a chromatin-permissive status that can influence neurogenesis and neuronal survival (Attia et al., 2007). *NAPB* is the large subunit of *NAP* and is related to growth inhibition by nitrite. Growth inhibition is attributed to excessive *NAPB* induced by nitrite (Jin et al., 2016). *NCOA7* is an essential V-ATPase modulator protein in the brain, regulating lysosomal function, neuronal junctions, and behavior (Castroflorio et al., 2021). Serine incorporator 1 (*SERINC1*) is a presumed carrier protein that promotes the synthesis of serine-derived lipids, which is essential for the function of some immune cells and does not contribute to the connection previously reported between lipid composition and autoimmunity in immune cells. The aforementioned literature implies that nine pyroptosis immune signatures serve a crucial role in the development and progression of glioma, providing a bridge to investigate the link between pyroptosis and the immune.

In this research, we constructed PIPM and discovered that the patterns of pyroptosis immune modification were related to several characteristics (Immunotherapy and radio-chemotherapy). Immunotherapy, epitomized by ICB (PD-1/L1), is a developmental area. However, the majority of patients have not achieved a prominent survival advantage, and immunotherapy is at a distance away from satisfying clinical anticipation (Xu et al., 2020). It is mainly due to the existence of inflammation in TIME, which hinders the infiltration and activation of immune cells. It has been found that the efficiency of immunotherapy can be significantly improved in the presence of radiochemotherapy-induced pyroptosis in TIME. This may improve the survival rate of patients compared to receiving ICB alone. Importantly, combination therapy can improve breast cancer cell sensitivity against PD- 1, which is due to pyroptosis-induced inflammation in the cancer-immune environment (Reck et al., 2019). Radiotherapy was the first of all intervention strategies to be proven beneficial in randomized controlled trials for glioma patients, and chemotherapy represented by TMZ was raised for the treatment of glioma in 2005. Radiotherapy and chemotherapy are employed as conventional adjuvant treatment and remarkably enhances the patients’ survival. Nevertheless, due to the presence of resistance mechanisms, such as those associated with TIME and stemness, most patients will develop treatment resistance (Rudà et al., 2018). From the above discussion, we found that pyroptosis is the key connection point in improving the efficacy of combined therapy.

The scoring strategy used for other kinds of cancer indicated a survival advantage of low PIPM risk score. To some extent, the PIPM risk score also reflects the aggression and invasiveness of cancers. The correlation between pyroptosis immune signatures, stemness, and PIPM risk score may reveal that both phenotypes are influenced by the pyroptosis immune in tumors, leading to uncontrolled immune turbulence and dedifferentiation defined by the loss of structure of origin (Du et al., 2020; Du et al., 2021).

Most previous literatures only evaluated only the perspective of pyroptosis genes, and only molecular typing by pyroptosis genes was performed to assess immune infiltration, without adding specific immune groups for analysis. Furthermore, pyroptosis is closely related to immunity and can activate tumor immunity to inhibit tumor growth (Zhang et al., 2020; Loveless et al., 2021), we are the first study to analyze the relationship between pyroptosis and immunity from a unique and direct perspective, obtaining four pyroptosis immune subgroups and nine pyroptosis immune signatures. PIPM constructed based on these nine signatures is highly predictive of prognosis, immunotherapy, and chemotherapy for glioma patients, and these genes can also be used as potential targets for later targeted therapy.

As far as we know, this is the forerunner research to perform a strategy for identifying the immune and pyroptosis features of pyroptosis immune modification patterns of glioma and to quantify the pyroptosis immune modification patterns using machine learning algorithms. This research presents a more complete knowledge of the TIME of glioma and develops a robust prognostic prediction model. However, three main shortcomings need to be further investigated. The first shortcoming, namely, due to the need to match multi-omics data with clinical information, we were confined to data from the TCGA and CGGA portals and could not cover other data sources. This hampered our ability to check the reliability of the model when applied with other data. The second shortcoming was that three omics data, comprising RNA-seq, mutation, and DNA methylation array data, are needed for the application of the prognostic prediction model, which is costly and difficult to implement in practical applications. Even so, rapid advances in biotechnology are expected to yield a three-in-one toolkit that will open the way for its implementation and generalization. Although multiple independent intrinsic validations were executed in this research, it was hardly possible to encompass all differences among patients from various geographical areas when tissues and data were retrospectively gathered in published databases. Finally, given that the microenvironmental features of different tumor regions may be distinct, such as tumor core and invasive margin, samples utilized for analyses were all obtained from the tumor core, and it is not possible to assess the pyroptosis and immune status in distinct tumor areas. Therefore, the results of this study await further validation by well-designed, farseeing, multi-center studies. However, despite such limitations, it is undeniable that our research offers important clues to elucidate the TIME of glioma.

## Conclusion

Pyroptosis serves an essential role in tumor suppression by stimulating antitumor immune responses. Meanwhile, the pyroptosis and immune status in TME of patients with glioma are strongly associated with prognosis. This pyroptosis immune signature mapping multi-omics data and clinical information can offer promising capabilities for risk classification and provide extra value beyond the single RNA_seq prognostic signature. Furthermore, PIPM has the potential to demonstrate compelling clinical value, potentially leading to the improved OS and even the evolvement of new treatment strategies for glioma patients.

## Data Availability

Publicly available datasets were analyzed in this study. This data can be found here: The R code has been uploaded to GitHub. This is the URL: https://github.com/shuaima1991/Pyroptosis-immune.git.
