# Supplementary material for: Extended Application of Genomic Selection to Screen Multi-Omics Data for the Development of Novel Pyroptosis-Immune Signatures and Predicting Immunotherapy of Glioma
Source: Front Pharmacol. 2022 May 10;13:893160. doi: 10.3389/fphar.2022.893160 (PMC9127445; doi:10.3389/fphar.2022.893160)
Supplement: Supplementary file 1 [file DataSheet3.docx]

The R code has been uploaded to GitHub. This is the URL: **https://github.com/shuaima1991/Pyroptosis-immune.git.**
