# Supplementary material for: Extended Application of Genomic Selection to Screen Multi-Omics Data for the Development of Novel Pyroptosis-Immune Signatures and Predicting Immunotherapy of Glioma
Source: Front Pharmacol. 2022 May 10;13:893160. doi: 10.3389/fphar.2022.893160 (PMC9127445; doi:10.3389/fphar.2022.893160)
Supplement: Supplementary file 2 [file DataSheet1.docx]

# Supplementary Materials

## Supplementary Figures

**Figure S1.** Overview of this work.


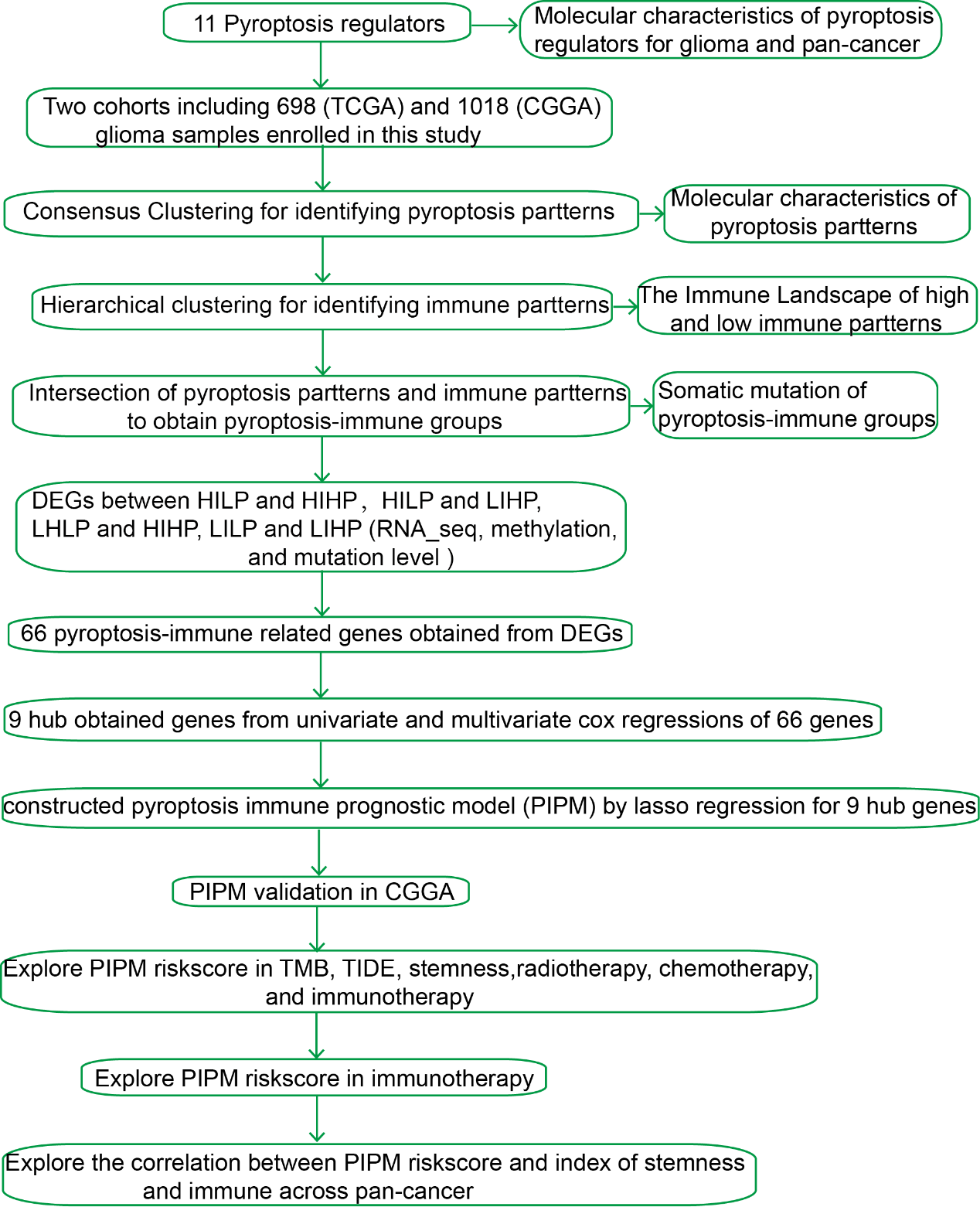


**Figure S2.** The Mutation characteristics of pyroptosis regulators in glioma and pan-cancer.


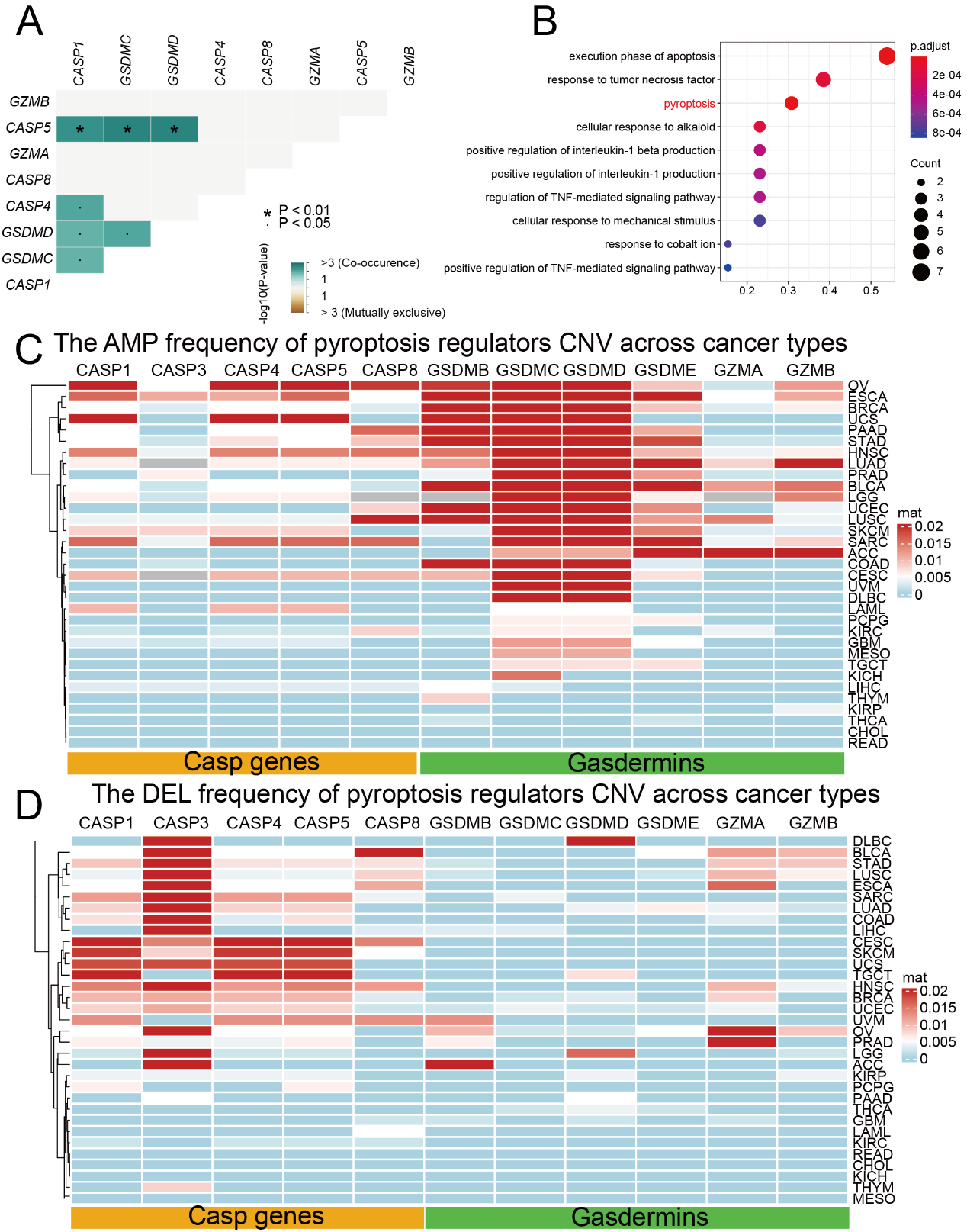


**(A)** The mutation co-occurrence and exclusion analyses for 11 pyroptosis-immune-related regulators. Co-occurrence, green; Exclusion, brown. **(B)** Gene Ontology (GO) enrichment analysis of 11 pyroptosis-immune-related regulators. **(C)** The CNV AMP alteration frequency of pyroptosis regulators across cancer types. **(D)** The CNV DEL alteration frequency of pyroptosis regulators across cancer types

**Figure S3.** The immune landscape of 698 glioma samples by hierarchical clustering.


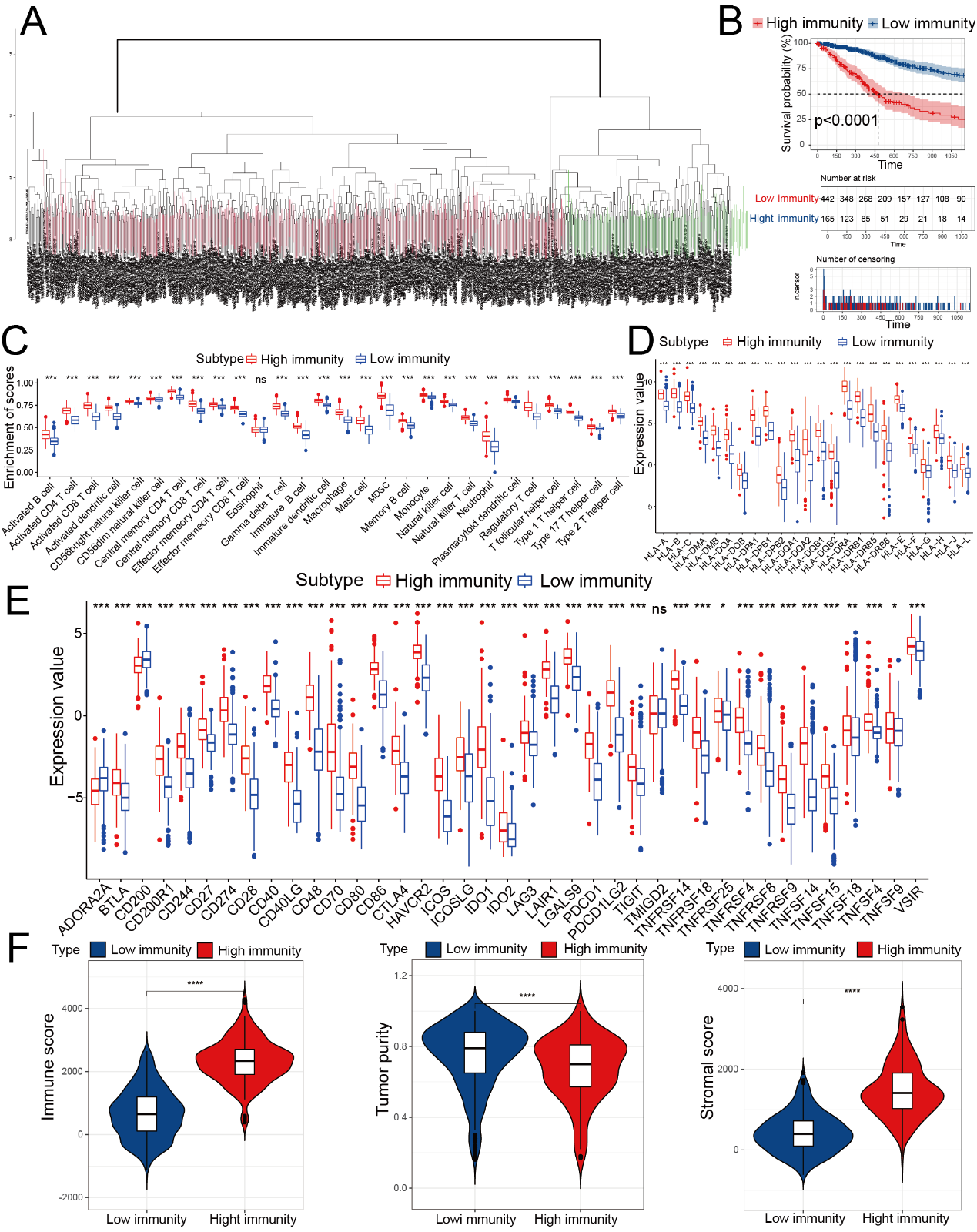


**(A)** The classification of 2 clusters. **(B)** KM curves for patients in high and low immunity groups. **(C)** The proportion of immune cells and in the two immunity clusters. **(D and E)** Differential expression of ICP genes and human leukocyte antigen (HLA) molecules among the glioma immune subtypes in TCGA cohorts. **(F)** The relationship between 2 clusters and ESTIMATEscore.

**Figure S4.** Four subgroups mutated differential genes.


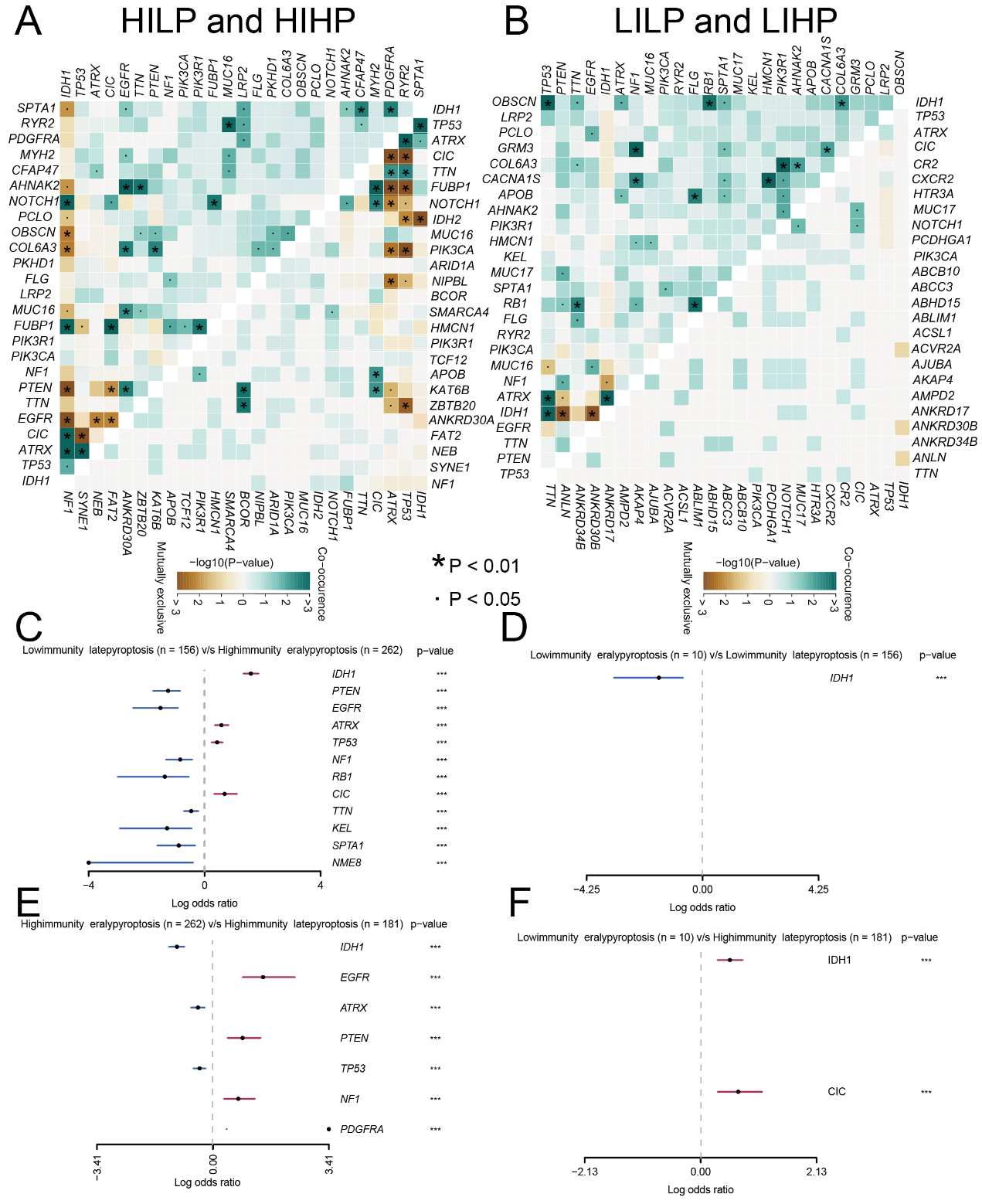


**(A and B)** The heatmap illustrates the mutually co-occurring and exclusive mutations of the top 25 frequently mutated genes. The color and symbol in each cell represent the statistical significance of the exclusivity or co-occurrence for each pair of genes. **(C-F)** Forest plot displays the top 10 most significantly differentially mutated genes between four cohorts.

**Figure S5.** the relationship between TMB mutation type of pyroptosis-immune–related groups.


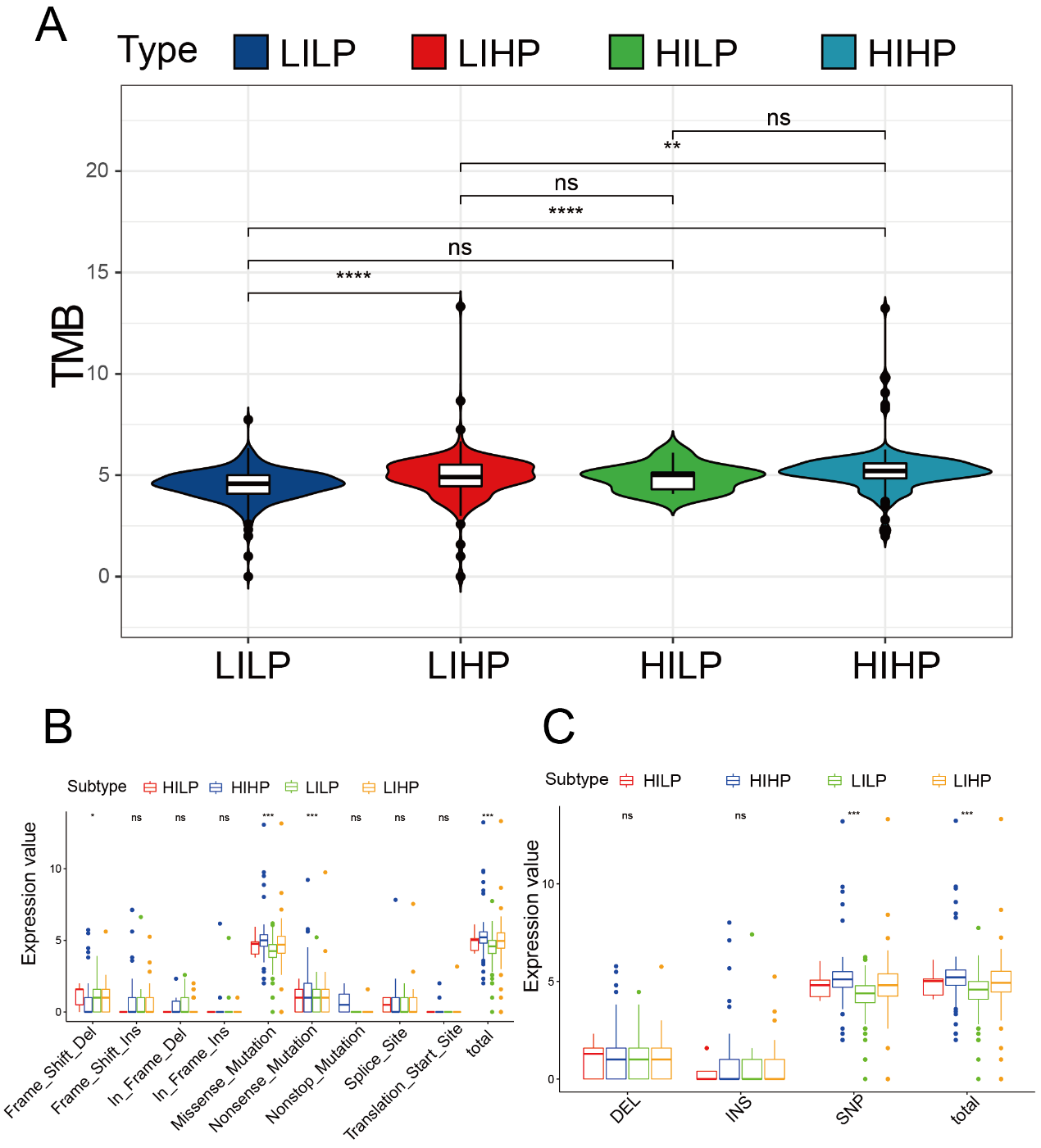


**(A)** Quantitative analysis of the proportion of TMB in the pyroptosis-immune–related groups. **(B and C)** Boxplots showing the comparisons of mutation frequencies of (A) every mutation type classified by effects (b), INDEL and SNP (c).

**Figure S6.** Distribution of four-group differentially expression genes (DEGs).


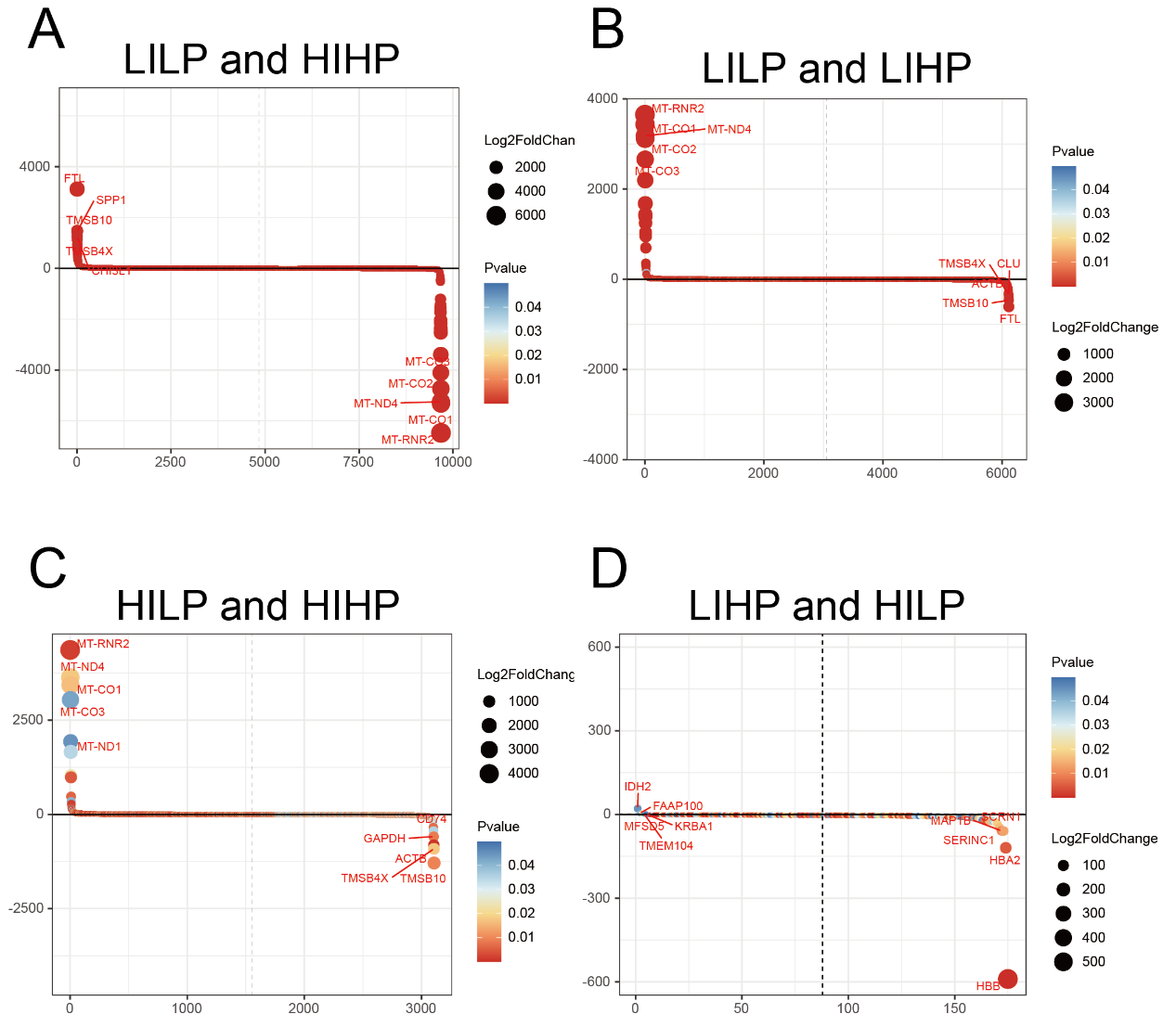


**(A-D)** Distribution of DEGs for “HILP and HIHP, HILP and LIHP, LHLP and HIHP, LILP and LIHP”.

**Figure S7.** The correlation between risk score and immune checkpoint molecules.


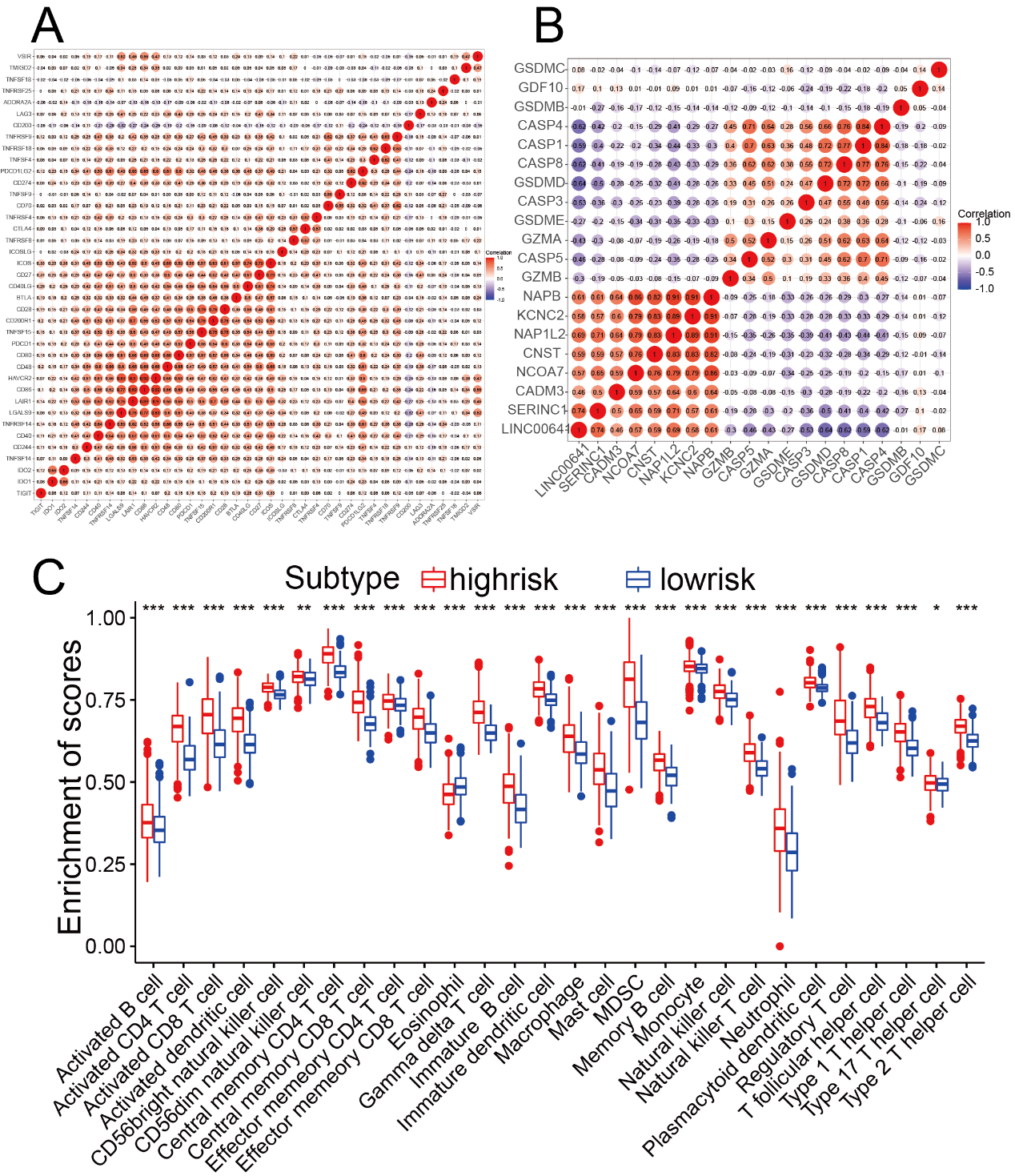


**(A)** The Pearson correlation coefficient calculated between risk score and nine immune

checkpoint molecules.

**(B)** The Pearson correlation coefficient calculated between pyroptosis genes and pyroptosis-immune related genes.

**(C)** Quantitative analysis of the proportion of 28 immune cells in high and low risk groups in the TCGA cohort.

**Figure S8.** Predicting patient prognosis in the TCGA cohort based on PIPM.


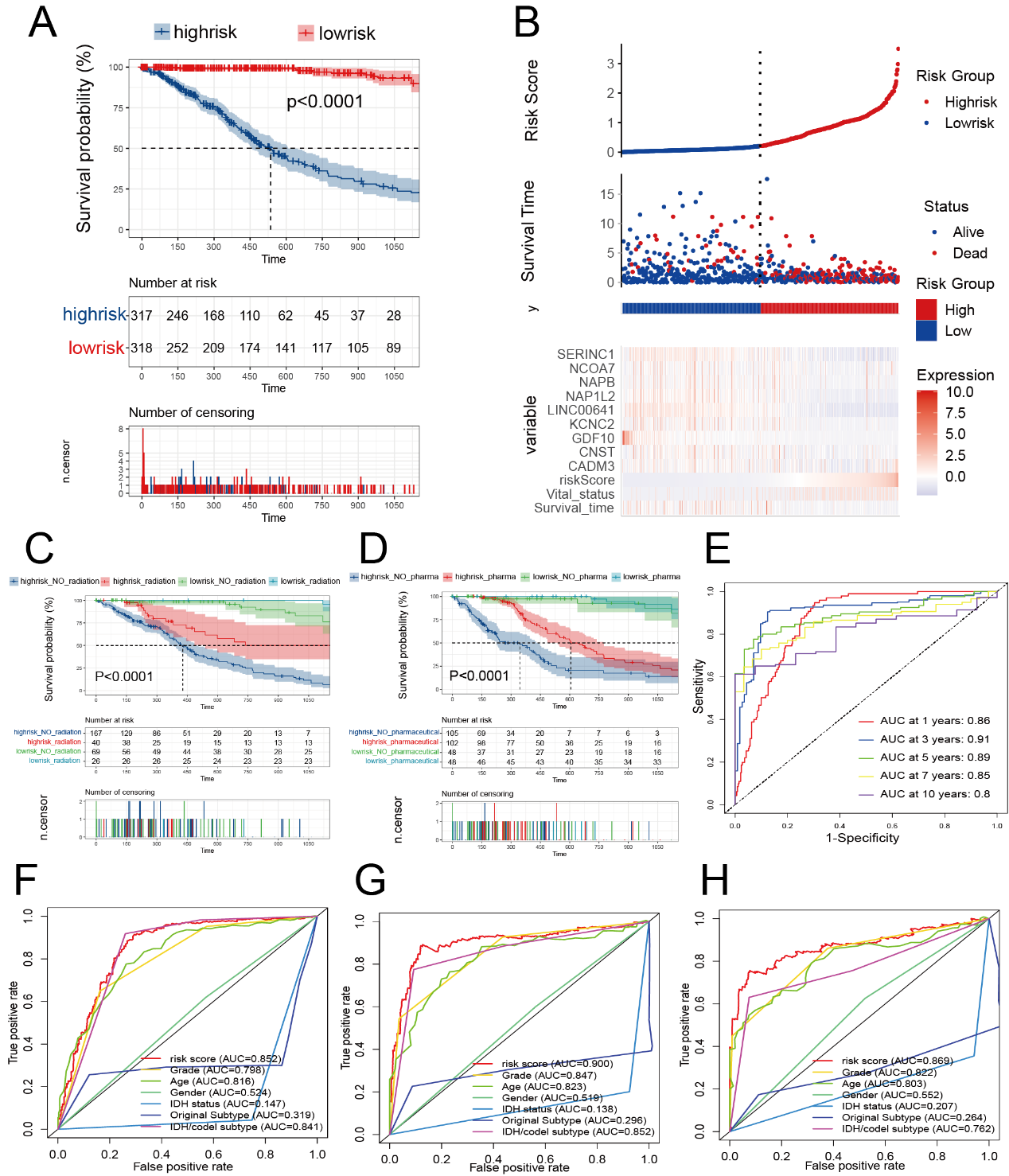


**(A)** KM curves for patients in high and lowrisk subgroups. **(B)** Patients were divided into highrisk and lowrisk subgroups based on median level of PIPM riskscore in TCGA cohort; heatmap of 9 prognostic immune regulators; survival status of patients in two subgroups. **(C,D)** KM curves for subgroup patients classified by both PIPM riskscore and therapy with radiotherapy (c) and pharmacological chemotherapy (d) in the TCGA cohort. **(E)** the timeROC curve to evaluate the prognostic value of PIPM riskscore in TCGA cohort. **(F-H)** the survivalROC curve to assess the prognostic value of PIPM riskscore and clinical features in TCGA cohort (1 year, 3 year, 5 year).

**Figure S9.** The relationship between grades, IDH1 mutation types, 1p19q status, and riskscore.


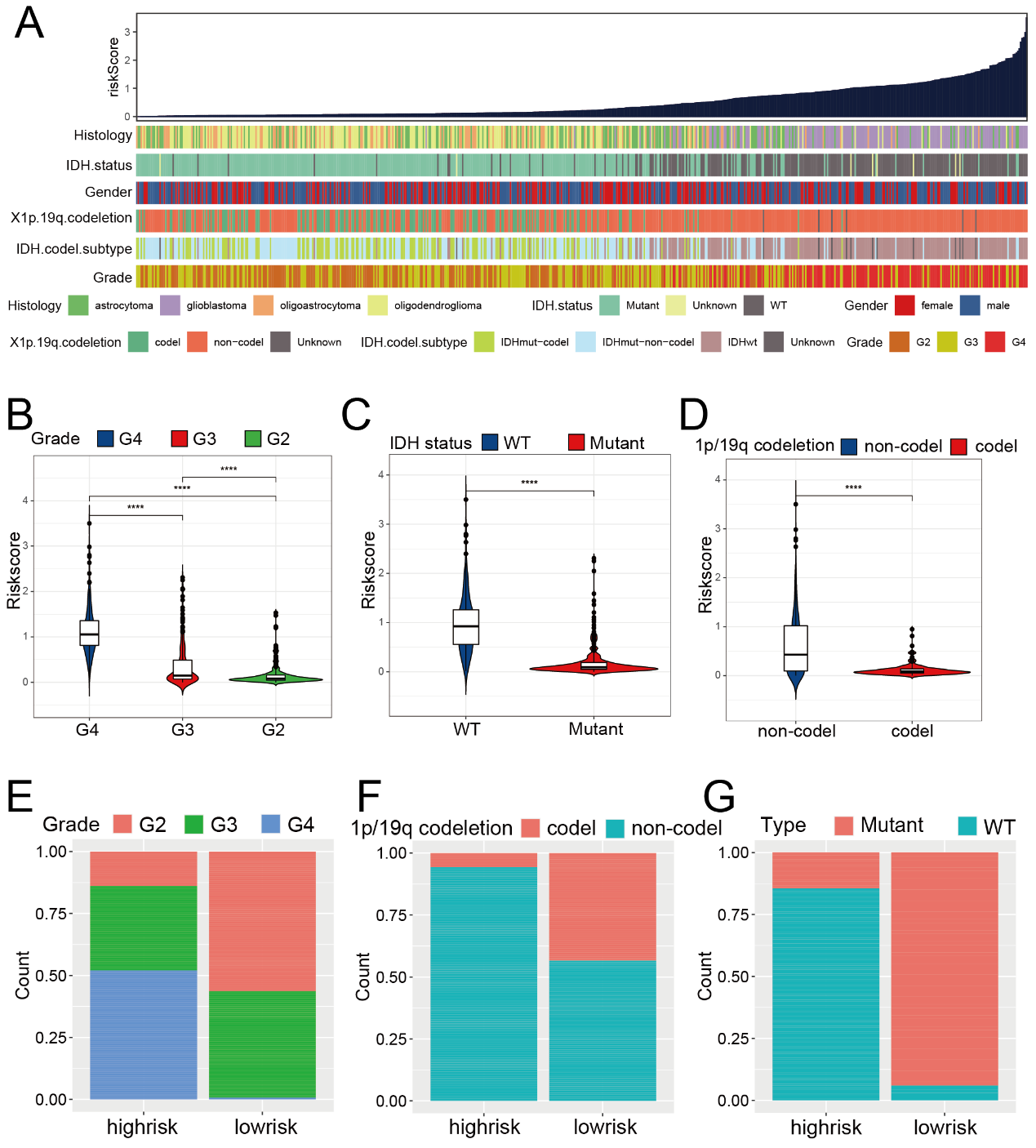


**(A)** An overview of the association between risksocre and clinical characteristics (Histology, IDH.status, Gender, IDHcodel.subtype and Grade). **(B-D)** Analyses of the relationship between IDH1 mutation type, recurrence status, 1p19q status and PIPM riskscore. **(E-G)** The proportion of patients with IDH1 mutation type, recurrence status, 1p19q status in the high or low-riskscore groups in the TCGA cohort.

**Fiugre S10.** Validation of PIPM in CGGA cohort.


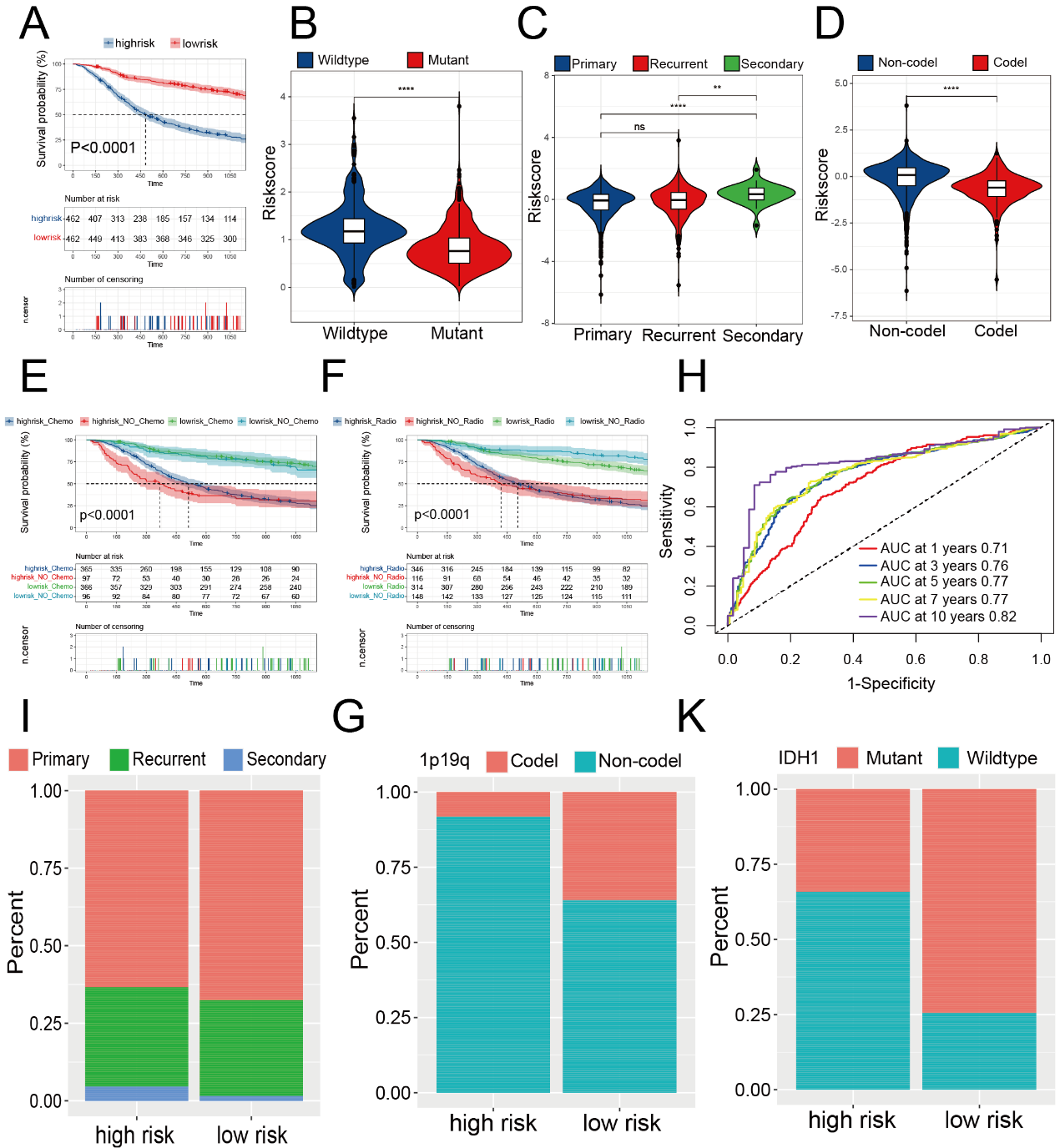


**(A)** KM curves for the PIPM-riskscore with the cut-off value 1018 of samples in the CGGA cohort. **(B-D)** Analyses of relationship between IDH1 mutation type, recurrence status, 1p19q status and PIPM riskscore. **(E,F)** Survival analyses for subgroup patients stratified by both PIPM riskscore and treatment with radiotherapy (e) and chemotherapy (f) in the CGGA cohort. **(H)** the timeROC curve to evaluate the prognostic value of risk score in the CGGA cohort. **(I-K)** The proportion of patients with IDH1 mutation type, recurrence status, 1p19q status in the high or low-riskscore groups in the CGGA cohort.

**Fiugre S11.** Functional enrichment analyses of DEGs between high-risk and low-risk group in the TGCA cohort.


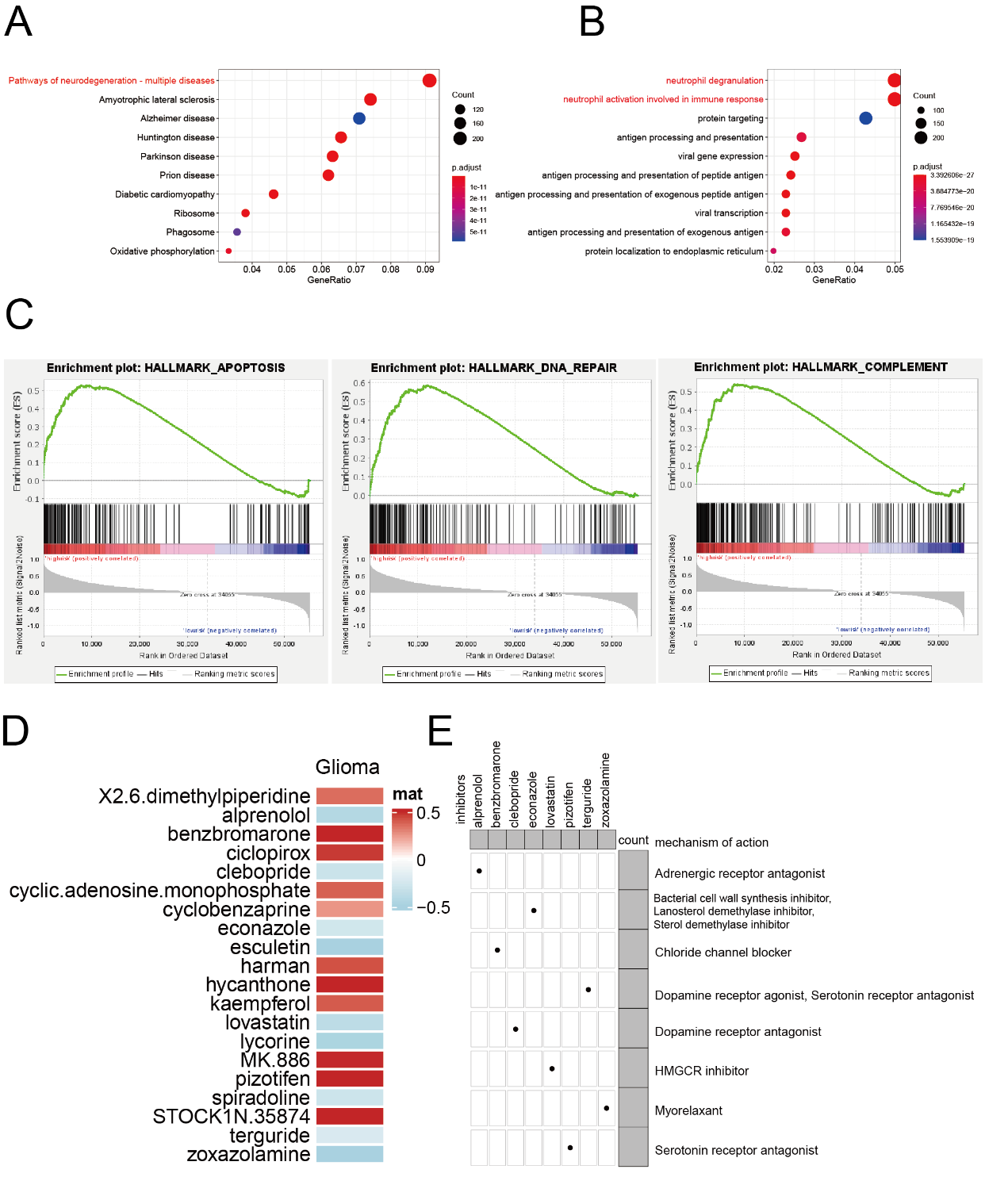


**(A and B)** Gene Ontology (GO) and Kyoto Encyclopedia of Genes and Genomes (KEGG) enrichment analysis of DEGs. **(C)** Gene set enrichment analysis (GSEA) enrichment analysis of DEGs. **(D)** Heatmap showing enrichment score of each compound from the CMap for glioma. Compounds are sorted from right to left by descending number of glioma significantly enriched. **(E)** Heatmap showing each compound (perturbagen) from the CMap that shares mechanisms of action (rows) and sorted by descending number of compound with shared mechanisms of action.

**Fiugre S12.** Analyses of relationship between TMB, TIS, TIDE and risk score.


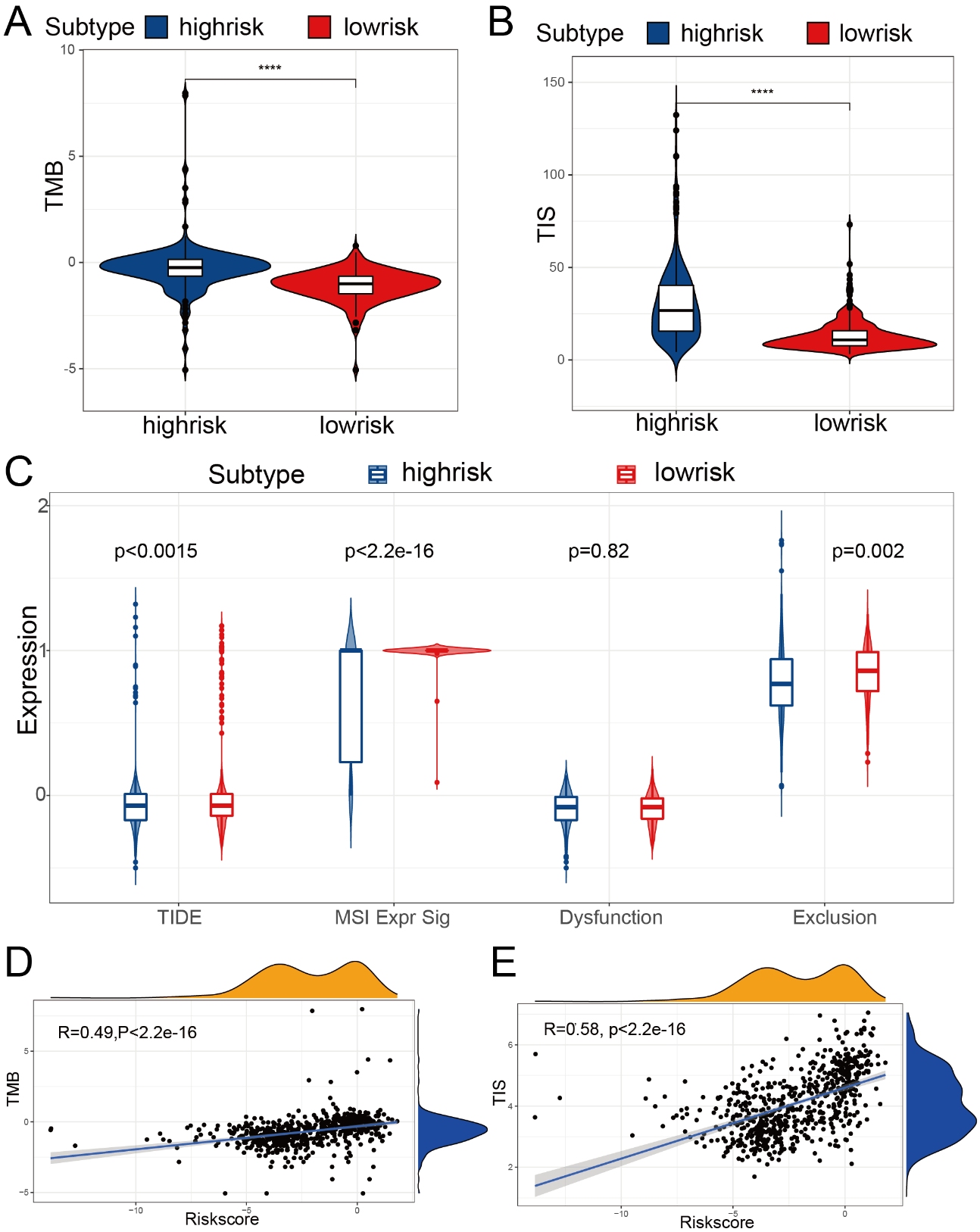
 **(A-C)** Relationship between TMB, TIS, TIDE and high and low risk groups. **(D-E)** Relationship between TMB, TIS, and riskscore.

**Fiugre S13.** Differences in stemness indices between the high and low risk groups.


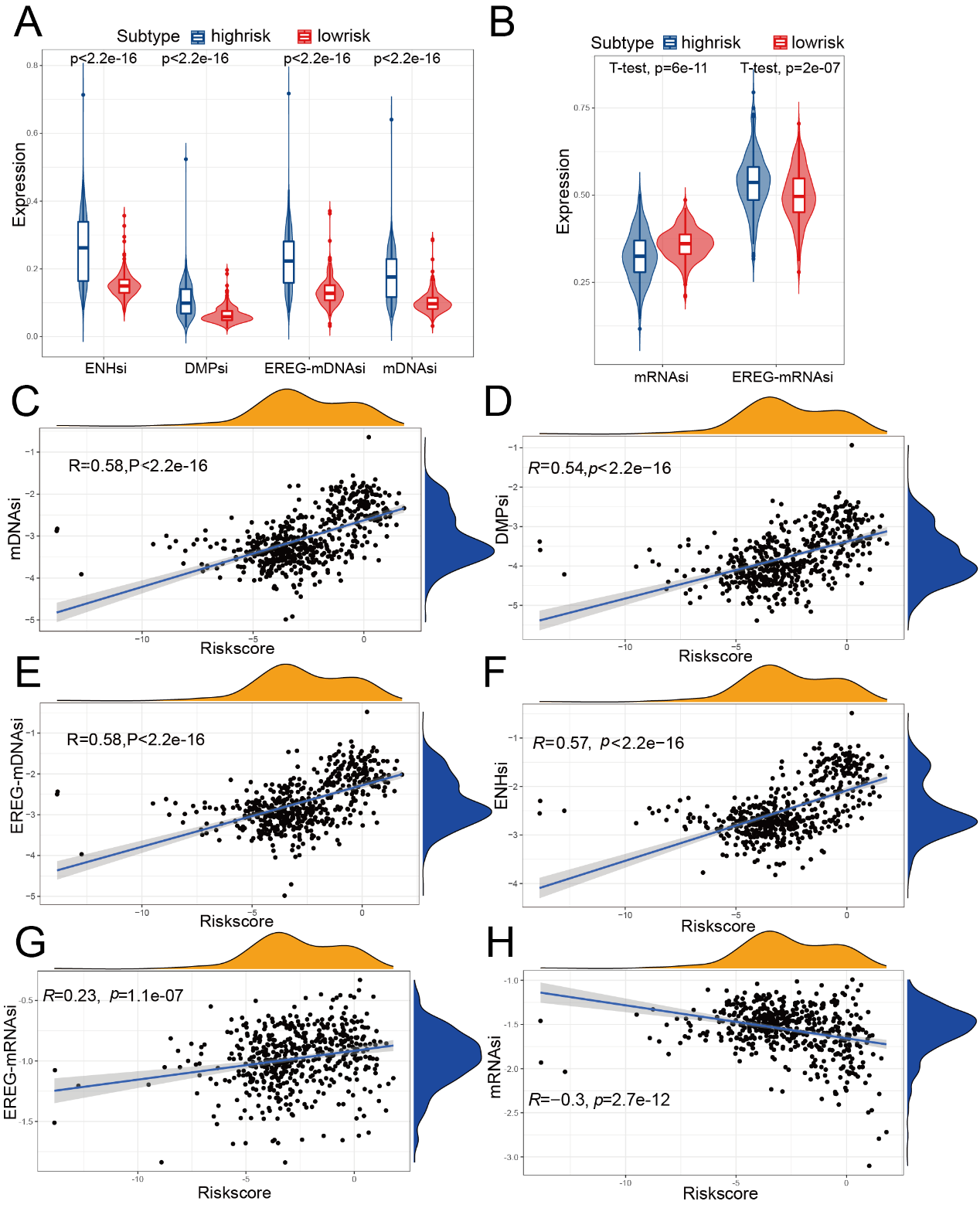
 **(A and B)** Analyses of relationship between stemness indices and high and low risk groups. **(C-H)** The relationship between mDNAsi, DMPsi, EREG-mDNAsi, ENHsi, EREG-RNAsi, mRNAsi and riskscore.

**Fiugre S14.** The landscape of PIPM in TCGA cohort.


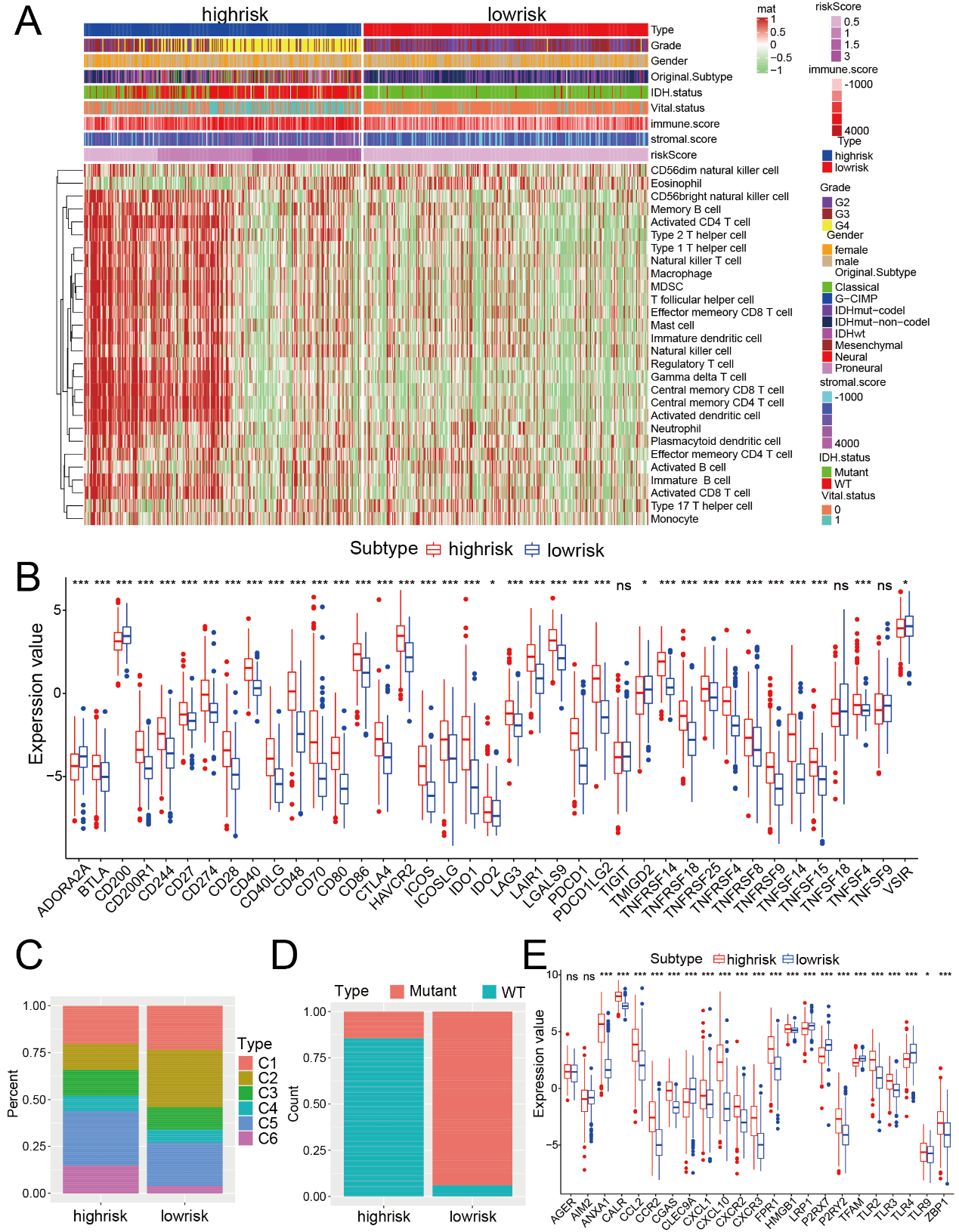
 **(A)** Heatmap of PIPM with 28 immune cells. **(B)** Differential expression of ICP genes among the PIPM in TCGA cohorts. **(C and D)**The proportion of patients with immune phenotypes (c) and IDH1 mutation type (d) in the high or low riskscore groups in the CGGA cohort. **(E)** Differential expression of ICD genes among the PIPM in TCGA cohorts.

**Figure S15.** The Immune Landscape of PIPM in the CCGA cohort.


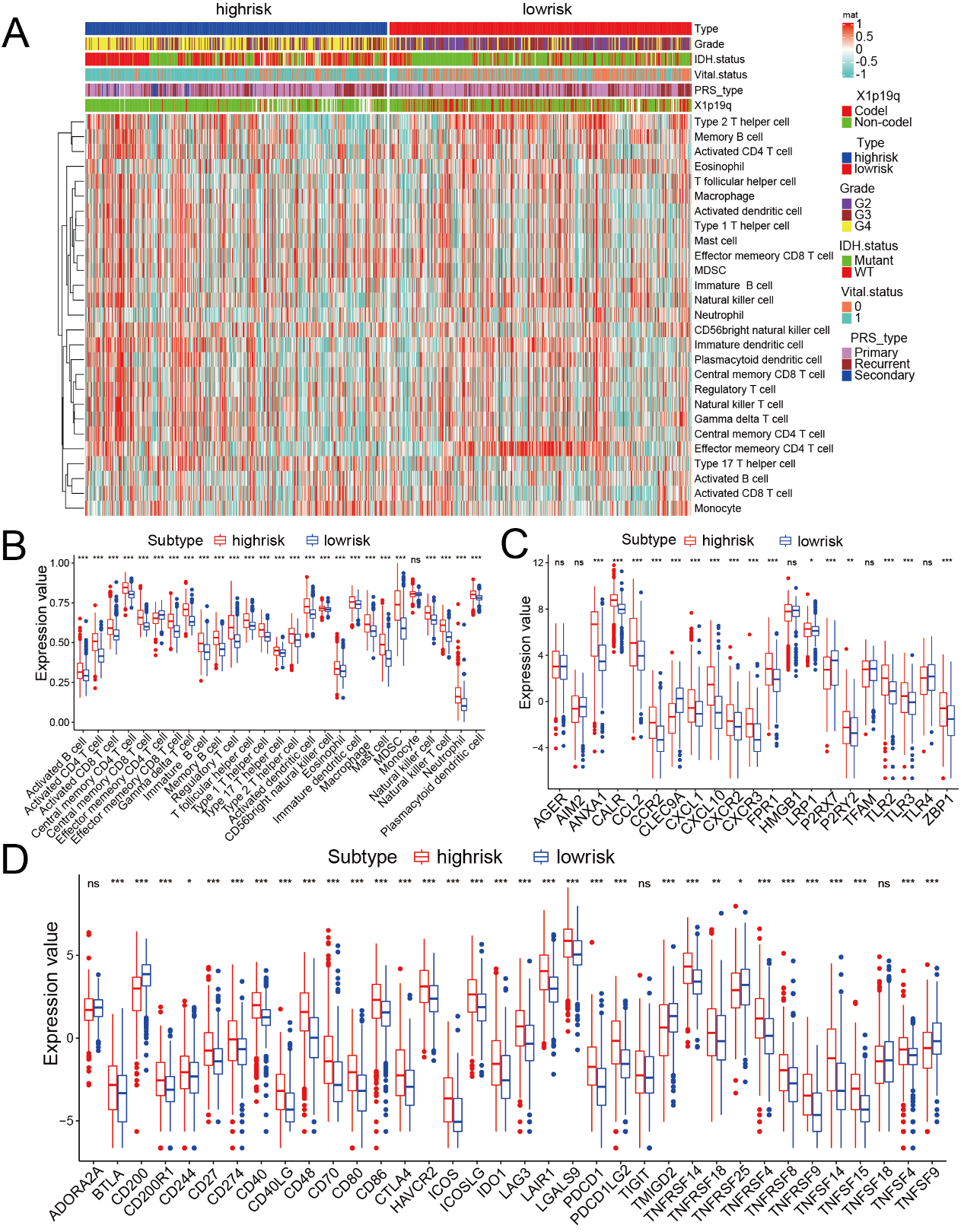


**(A)** Heatmap of high and low risk group with immune cell infiltration. **(B)** Quantitative analysis of the proportion of 28 immune cells in high and low risk groups in the CCGA cohort. **(C)** Differential expression of ICP genes between high and low risk group in TCGA cohorts. **(D)** Distribution of ICD genes between high and low risk group in the TCGA cohorts.

**Figure S16** The robust role of the PIPM in PD1/PD-L1 immunotherapy.


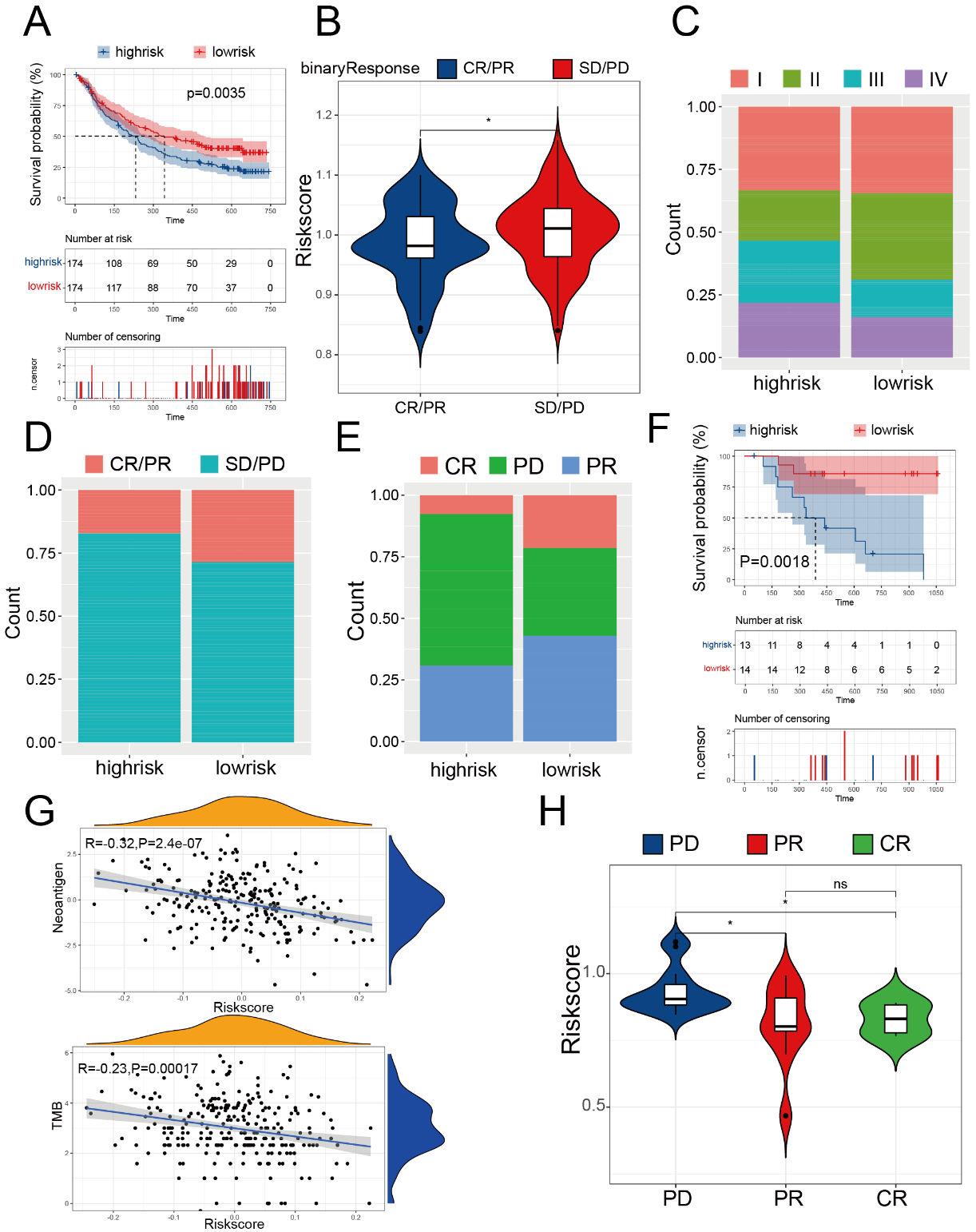


**(A)** KM curves for subgroup patients classified by both PIPM riskscore in IMvigor210 cohort. **(B)** The proportion of PIPM risk scores in the CR/PR and SD/PD groups in the IMvigor210 group. **(C)** The proportion of patients in the IMvigor210CoreBiologies cohort with clinical grades in the high or low-PIPMscore groups. **(D)** The percentage of patients in the IMvigor210CoreBiologies cohort with clinical response in the high or low-PIPMscore groups. **(E)** The percentage of patients in the GSE78220 cohort with clinical response in the high or low PIPM riskscore groups. **(F)** KM curves for subgroup patients classified by both PIPM riskscore in GSE78220 cohort. **(G)** The relationship between Neoantigen, TMB and PIPM riskscore. **(H)** Different PIPM-score in PD, PR, and CR groups in GSE78220 cohort.

**Figure S17.** Survival of PIPM riskscore across tumor types.


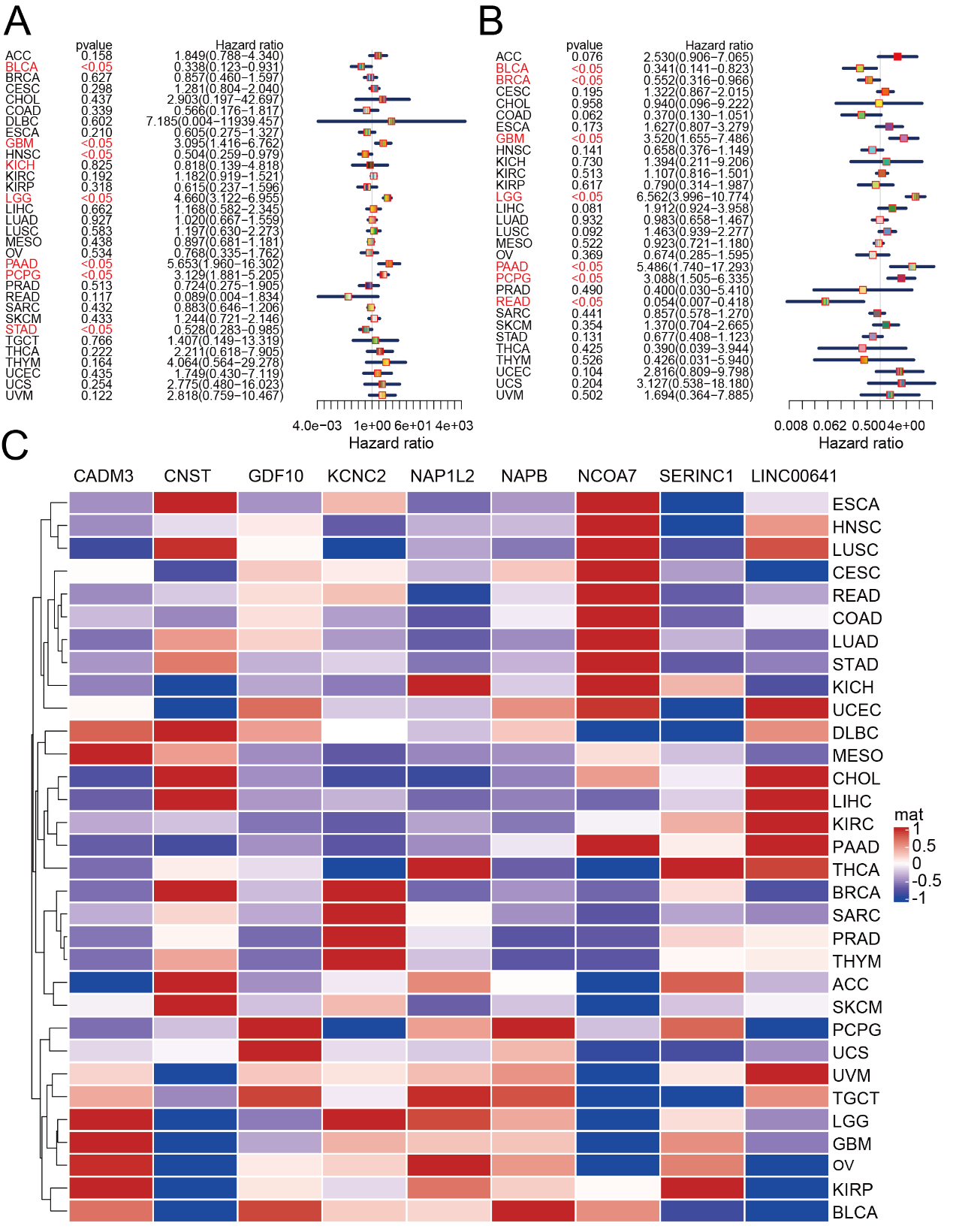


**(A and B)** The prognosis-free interval (a) and overall survival (b) analyses for the PIPM riskscore in TCGA cancer types using a univariate Cox regression model. Hazard ratio>1 represented risk factors for survival and hazard ratio<1 represented protective factors for survival. **(C)** The expression level of pyroptosis-immune related genes across tumor types.
